# Supplementary material for: Multichannel multicentroid motion-compensated single pixel imaging of a 2D arbitrarily moving rigid-body target
Source: Commun Eng. 2026 Feb 25;5:61. doi: 10.1038/s44172-026-00619-2 (PMC13046804; doi:10.1038/s44172-026-00619-2)
Supplement: Supplementary file 2 — Supplementary Information [file 44172_2026_619_MOESM2_ESM.pdf]

Multichannel multicentroid motion-compensated single-pixel imaging of an 2D arbitrarily moving rigid-body target Supplementary material

Chongwu Shao, Yue Cao, Shijian Li*, Xuri Yao* and Qing zhao*

Supplementary Note 1: Optimized Fourier localization method

Fourier transform is essentially a mapping from the global to the point, where each coefficient in the Fourier domain is contributed by all points in the spatial domain. This implies that a change at any point in the spatial domain induces variations in all coefficients of the Fourier domain. Specifically, a displacement of the target object in the spatial domain corresponds to a phase shifting in the frequency domain[1]. Based on this characteristic, we can detect the presence or movement of objects in a scene by monitoring the phase changes of specific coefficients in the Fourier domain. Fourier-SPI implements this by modulating light with Fourier basis patterns to directly measure specific Fourier coefficients.

Specifically, we exploit the Spatial-Shift property of the Fourier transform, whereby a spatial translation corresponds to a phase modulation in the frequency domain. As shown in the following:

Let $\mathcal{F}\{f(x)\}(\xi) = F(\xi) = \int_{-\infty}^{\infty} f(x)\, e^{-j2\pi\xi x}\, dx$. For any $x_0 \in \mathbb{R}$,

$$\mathcal{F}\{f(x-x_0)\}(\xi) = \int_{-\infty}^{\infty} f(x-x_0)e^{-j2\pi\xi x}\, dx = e^{-j2\pi\xi x_0} \int_{-\infty}^{\infty} f(t)e^{-j2\pi\xi t}\, dt = e^{-j2\pi\xi x_0}\, F(\xi). \tag{1}$$

The 2D and DFT cases follow analogously, for any $x_0, y_0 \in \mathbb{N}$,

$$\mathcal{F}\{f(x-x_0, y-y_0)\}(u,v) = \sum_{x=0}^{N-1} \sum_{y=0}^{M-1} f(x-x_0, y-y_0)e^{-j2\pi(ux+vy)} \tag{2}$$

For a target smaller than the field of view, its translation can be regarded as a circular shift of the entire array. With the cyclic substitution $x' = x - x_0$, $y' = y - y_0$, the mapping is a bijection on $\{0, \ldots, M-1\} \times \{0, \ldots, N-1\}$, therefore, any discrete sum over this index set remains invariant.

$$\mathcal{F}\{f(x-x_0, y-y_0)\}(u,v) = \sum_{x'=0}^{N-1} \sum_{y'=0}^{M-1} f(x', y')e^{-j2\pi(u(x'+x_0)+v(y'+y_0))} = e^{-j2\pi(ux_0+vy_0)}F(u,v). \tag{3}$$

It is noteworthy that the minimum sampling unit in SPI is a single pixel. Accordingly, a sub-pixel displacement of the target induces an energy redistribution across neighboring samples at the resolution of the SPI sampling grid, which should be regarded as a nonrigid apparent displacement rather than a strict translation. To robustly capture such minute displacements, increasing the SPI sampling-grid resolution is the most reliable strategy.

As a result, with the Fourier basis pattern:

$$P(x, y \mid f_x, f_y) = \exp\left[-2\pi j \cdot (f_x x + f_y y)\right], \tag{4}$$

a displacement (Δx, Δy) in the spatial domain therefore induces a phase shift ($-2\pi f_x \Delta x$, $-2\pi f_y \Delta y$) in the Fourier domain of the pattern. In practice, we employ two sets of patterns, setting $f_x = 0$ and $f_y = 0$, respectively, to determine the relative displacement Δy and Δx of the target.

Absolute-position localization method

MC3-SPI requires determining the absolute positions of multiple centroids on the target to achieve pose sensing. Consequently, our localization method must be capable of pinpointing the absolute coordinates of these centroids.

We use the following Fourier basis patterns with spatial frequencies f to localize the absolute centroids of the target.

$$\begin{aligned} P_x &= P(x, y \mid f, 0) = \exp\left(-2\pi j\, f\, x\right), \\ P_y &= P(x, y \mid 0, f) = \exp\left(-2\pi j\, f\, y\right). \end{aligned} \tag{5}$$

We expect the phase of the Fourier coefficients (φ_x, φ_y) to correspond linearly and directly to the target's coordinates (x, y).

$$x = \frac{-\varphi_x}{2\pi f}, \quad y = \frac{-\varphi_y}{2\pi f}. \tag{6}$$

As shown above, we have established an absolute position localization model based on Fourier basis patterns. Therefore, we need to determine an appropriate spatial frequency f to achieve the best localization accuracy.

We first examine the periodic structure of the Fourier basis patterns. From Eq.3 it follows that:

$$\mathcal{F}\{o(x + 1/f, y)\}(f, 0) = e^{-j2\pi(f \cdot 1/f)} \mathcal{F}\{o(x, y)\}(f, 0) = \mathcal{F}\{o(x, y)\}(f, 0). \tag{7}$$

This implies that two identical targets separated by a relative displacement of $1/f$ are indistinguishable. Equivalently, we require $1/f \geq M$, where the field of view (FOV) is $M \times M$ pixels. Intuitively, when Fourier basis patterns with spatial frequency $f > 1/M$ are used for localization, the modulation patterns in SPI exhibit a periodic distribution, which makes it impossible to identify the specific period in which the target resides for absolute localization.

Therefore, absolute localization is meaningful only when the spatial frequency satisfies $f \leq 1/M$. Accordingly, all phases considered henceforth are restricted to the range $[0, 2\pi)$.

With an appropriate configuration of the Fourier basis patterns, the absolute positions of small targets can be approximately localized[2]. Through the translation property of the Fourier transform, it is evident that a target's displacement in the spatial domain corresponds linearly to the phase shift of its Fourier transform coefficients, with the proportionality constant being the frequency of the Fourier coefficient. Therefore, it suffices to consider localization at an arbitrary position.

We first note that for any single pixel $\delta(x_0, y_0)$, our Fourier localization method is exact.

$$\langle \delta(x_0, y_0), P_x \rangle = \sum_{x=0}^{N-1} \sum_{y=0}^{M-1} \delta(x_0, y_0) e^{-j2\pi f x} = e^{-j2\pi f x_0} \tag{8}$$

The direction y is identical to the direction x. For simplicity, we reduce the above expression to the following form.

$$\langle \delta(x_0, y_0), P_x \rangle = e^{j\varphi_0}, \quad \varphi_0 = -2\pi f x_0. \tag{9}$$

Consider a complex target $\sum_{i=0}^{n-1} A_i\delta(x_i, y_i)$, where a single pixel $A_i\delta(x_i, y_i)$ corresponds to φ_i. The localization result is given by the expression below:

$$\left\langle \sum_{i=0}^{n-1} A_i\delta(x_i, y_i), P_x \right\rangle = \sum_{i=0}^{n-1} A_i e^{j\varphi_i} = Ae^{j\varphi}, \quad \varphi_i = -2\pi f x_i. \tag{10}$$

In practice, the phase ψ corresponding to the correct localization result is $\frac{\sum_{i=0}^{n-1} A_i\varphi_i}{\sum_{i=0}^{n-1} A_i}$. The phases ψ and φ are evidently not identical; their difference is determined by the structure of the target, which we consider as an intrinsic phase $\Delta\Phi$. Using the shift invariance of the complex argument, $\arg\big(e^{-j\psi}Z\big) = \arg(Z) - \psi$, the explicit form of $\Delta\Phi$ is given by:

$$\Delta\Phi = \psi - \varphi = \arg\Big(\sum_{i=0}^{n-1} A_i e^{j(\varphi_i - \psi)}\Big). \tag{11}$$

The correct localization phase satisfies $\psi \in (\min(\varphi_i), \max(\varphi_i))$. Hence, the more concentrated the distribution of $\{\varphi_i\}$, the smaller the intrinsic phase $\Delta\Phi$. Because $\varphi_i = -2\pi f x_i$, reducing either the spatial frequency f or the object size narrows the spread of $\{\varphi_i\}$, thereby decreasing $\Delta\Phi$ and improving the accuracy of the absolute localization. Additionally, when the object is symmetric, $\Delta\Phi = 0$.

However, aside from the central zero frequency $f = 0$, the Fourier coefficients unavoidably possess the inherent phase $\Delta\Phi$, effectively an initial offset, which leads to systematic errors in determining the absolute position with Fourier basis patterns. To mitigate the impact of inherent phase on absolute position accuracy, we move beyond the discrete Fourier transform and adopt Fourier basis patterns with lower, non-integer spatial frequencies ($f = 1/M, 1/(2M), 1/(3M), ...$) for target modulation.

Numerical simulation of absolute-position localization

To quantitatively assess the impact of the frequency of the Fourier basis pattern on the absolute coordinate localization accuracy, we conducted numerical simulations. We tracked targets of varying sizes using Fourier basis patterns with spatial frequencies of $1/M$, $1/(2M)$, $1/(3M)$, and $1/(4M)$, respectively. The Fourier localization basis patterns were 256×256 pixels in size and no up-sampling was employed. For the targets, 500 random handwritten digits were selected from the MNIST dataset, each target positioned randomly 100 times to calculate the average localization RMSE.

As shown in Supplementary Fig.1(a), in agreement with our previous hypothesis, lower spatial frequencies f yield higher localization accuracy. Furthermore, regardless of the Fourier frequency, which is in agreement with our previous hypothesis either. These results confirm the validity of our theory.

When applying Fourier basis patterns in a DMD-based SPI system, it is necessary to employ a phase-shifting method to separate the basis patterns and to binarize the grayscale images. Using temporal dithering would significantly compromise the system's temporal resolution, which is unacceptable for high-speed target localization. To ensure sufficient temporal resolution, we adopt a three-step phase-shifting approach combined with a spatial dithering strategy that leverages the "spatial averaging effect"[3]. However, this binarization process sacrifices spatial resolution, resulting in localization errors. Moreover, because simulating grayscale requires a region of pixels, this error significantly affects the localization of small targets. It is essential to focus on the impact of spatial dithering on the localization error of Fourier basis patterns with different spatial frequencies.

We conducted further numerical simulations by introducing spatial dithering for binarization on the basis of Supplementary Fig.1(a). Using the conventional Floyd-Steinberg dithering method[4], the results are shown in Supplementary Fig.1(b). As mentioned earlier, errors caused by spatial dithering are particularly pronounced for small targets, whereas for larger targets, the errors primarily stem from the inherent phase of the Fourier coefficients.

In this context, we observe that lower spatial frequencies f lead to larger localization errors, especially when the target is small. This occurs because, as the spatial frequency f decreases, the grayscale

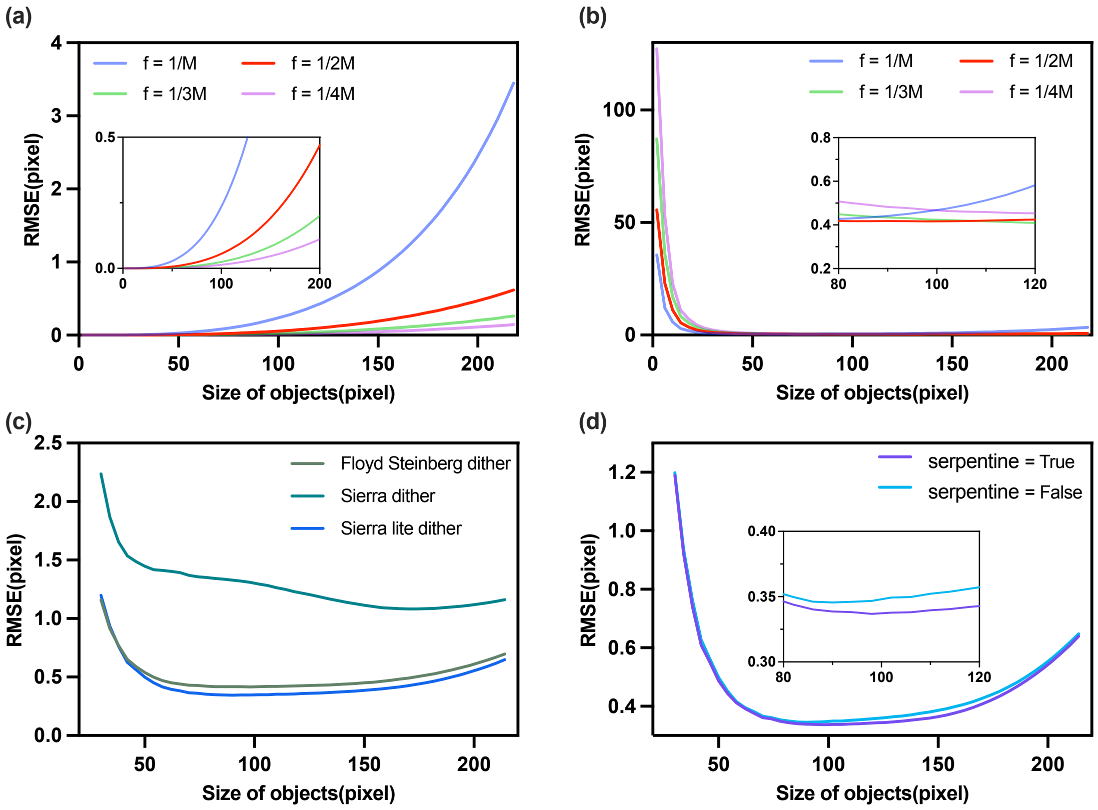

Supplementary Fig. 1: Performance analysis of the optimized Fourier localization method combined with various binarization techniques. (a) RMSE in target localization for different target sizes using Fourier basis patterns at various frequencies. (b) Under three-step phase shifting combined with the Floyd-Steinberg dithering method, RMSE in target localization for different target sizes using Fourier basis patterns at various frequencies. (c) Analysis of the localization RMSE for different target sizes using Fourier basis patterns with $f = 1/(2M)$ under three-step phase shifting, employing various dithering methods. (d) Analysis of the localization RMSE for different target sizes using Fourier basis patterns with $f = 1/(2M)$ under three-step phase shifting, utilizing the Serria-Lite dithering method along different error diffusion paths.

variation of the patterns becomes more gradual, requiring greater contrast to discriminate differences, which is unfavorable under spatial dither. As shown in Supplementary Fig.2, we present pattern images at different spatial frequencies with magnified details. After spatial dither, patterns with lower spatial frequencies f provide insufficient contrast to distinguish grayscale details, resulting in poorer localization accuracy.

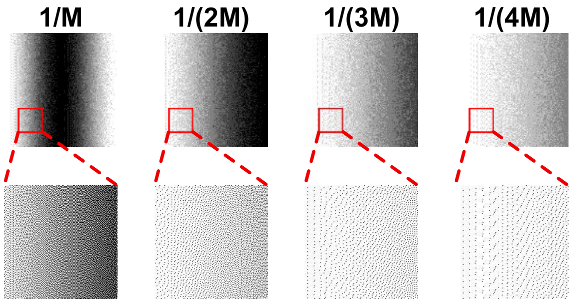

Supplementary Fig. 2: Real DMD-patterns obtained by applying spatial dither and binarization to Fourier basis patterns at different spatial frequencies. Local magnifications highlight details and provide a direct visual comparison of grayscale contrast under the spatial-dither simulation.

In summary, the presence of spatial dither causes lower spatial frequencies f to yield larger localization errors, which contradicts the theoretical expectation of the Fourier localization method that lower spatial frequencies f should produce smaller localization errors. Therefore, a trade-off must be made between the systematic error induced by the inherent phase $\Delta\Phi$ of the Fourier localization method and the quantization noise resulting from spatial dithering. Our numerical simulations reveal, as shown in the inset of the Supplementary Fig.1(b), that Fourier basis patterns with $f = 1/(2M)$ offer the best localization accuracy for typical target sizes (80 to 120 pixels), corresponding to approximately 1/9 to 1/4 of the FOV.

To further enhance the performance of the system, we investigated the impact of different spatial dithering strategies on the localization accuracy of Fourier basis patterns. The essence of spatial dithering for binarization lies in error diffusion, where the quantization error of a single pixel is spread to adjacent pixels. By leveraging the “spatial averaging effect", the local average quantization error approaches zero, thereby achieving an approximation of the grayscale distribution.

The kernel function $K(x, y)$ is a key parameter in error diffusion methods, and different dithering algorithms have different kernel functions. The Floyd-Steinberg kernel function is given as follows:

$$K(x,y) = \frac{1}{16}\begin{bmatrix} - & X & 7 \\ 3 & 5 & 1 \end{bmatrix}. \tag{12}$$

The Serria kernel function is given as follows:

$$K(x,y) = \frac{1}{32}\begin{bmatrix} - & - & X & 5 & 3 \\ 2 & 4 & 5 & 4 & 2 \\ 0 & 2 & 3 & 2 & 0 \end{bmatrix}. \tag{13}$$

The Serria-Lite kernel function is given as follows:

$$K(x,y) = \frac{1}{4}\begin{bmatrix} - & X & 2 \\ 1 & 1 & 0 \end{bmatrix}. \tag{14}$$

Where, “–” denotes pixels that have already been processed, X represents the pixel currently being processed, and the adjacent numbers (weights) indicate the proportion of the error allocated to that position.

Using the three error diffusion methods mentioned above for spatial dithering-based binarization, the numerical simulation results regarding their impact on localization accuracy are shown in Supplementary

Fig. 1(c). The Serria-Lite method provides the best accuracy. In contrast, the localization error using the Serria method with a larger kernel is significantly worse than that obtained with a smaller kernel. A similar conclusion is drawn in Fourier-SPI, where the Fourier-SPI based on the Serria-Lite method achieves the best image quality[5]. We believe that a smaller kernel requires a smaller region for the quantization error to average out to nearly zero, making it more suitable for capturing fine details of the target, which is beneficial for both detailed imaging and centroid localization.

To minimize quantization errors in binary Fourier patterns and enhance their structural fidelity to grayscale counterparts, we employ the Sierra Lite dithering kernel in conjunction with a serpentine scanning strategy: processing odd rows from left to right followed by even rows from right to left.

Therefore, the kernel function for the odd rows can be expressed as:

$$K_{\rightarrow}(x,y) = \frac{1}{4}\begin{bmatrix} - & X & 2 \\ 1 & 1 & 0 \end{bmatrix} \tag{15}$$

Consequently, the kernel function for the even rows is given by:

$$K_{\leftarrow}(x,y) = \frac{1}{4}\begin{bmatrix} 2 & X & - \\ 0 & 1 & 1 \end{bmatrix} \tag{16}$$

The serpentine diffusion path is shown below:

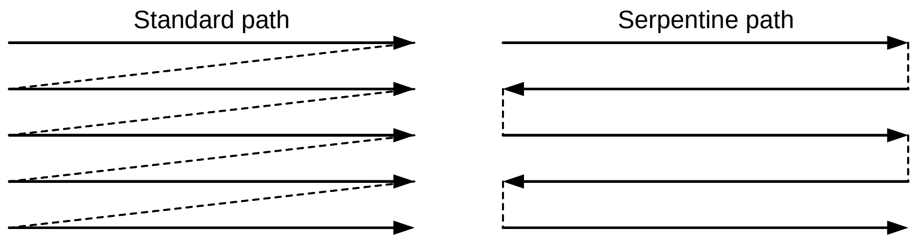

(17)

We also conducted numerical simulations, with the results shown in Supplementary Fig.1(d). The use of a serpentine error diffusion path optimizes overall localization accuracy, particularly within the typical target size range ($1/9 \sim 1/4$ of the FOV), successfully keeping the localization error below 0.35 pixels. Thus, the serpentine error diffusion path further enhances the localization accuracy of Fourier patterns.

In summary, we have implemented a Fourier localization method for absolute coordinate positioning in a DMD-based SPI system. Specifically, we achieved absolute coordinate localization in DMD-based SPI by employing Fourier basis patterns with $f = 1/(2M)$, a three-step phase-shifting approach, the Sierra-Lite dithering method and a serpentine error diffusion path. This method controls the localization error for typical target sizes to approximately 1/3 of a pixel.

Supplementary Note 2: Sampling strategy of MC3-SPI with Fourier patterns

Above all, it is worth noting that the Fourier spectrum of a static image is conjugately symmetric about the central zero frequency. Therefore, in conventional Fourier-SPI, we only need to acquire the upper half of the Fourier spectrum and then use the conjugate symmetry to recover the complete spectrum.

General sampling strategy

When the target is in motion, a detailed analysis is required, as illustrated in Supplementary Fig.3. When the target undergoes only translational movement, translation affects only the phase of the Fourier spectrum, and this phase can be corrected using the localization information. After compensation,

the conjugate Fourier patterns remain equivalent, allowing the conventional strategy to be employed. However, for a rotating target, its Fourier spectrum rotates according to the rotation angle. In such cases, the Fourier coefficients are obtained from conjugate Fourier patterns at different times for targets with varying rotation angles, unable to maintain conjugate. Therefore, when handling rotation, the conjugate acquisition modes in Fourier-SPI are not equivalent, which can provide more image information.

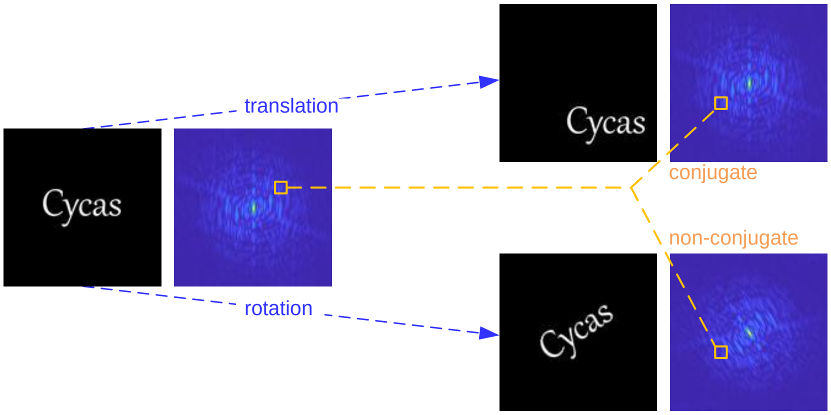

Supplementary Fig. 3: The Effect of Motion Patterns on the Fourier Spectrum.

Moreover, in Fourier-SPI, image information is concentrated in the low-frequency region naturally, meaning that low-frequency data carry far more weight than high-frequency data. Consequently, in our scheme, we employ a full Fourier spectrum sampling mode without omitting half of it, ensuring that sufficient low-frequency information is captured.

When designing a specific sampling strategy, one must fully account for the structural characteristics of the Fourier spectrum. In the Fourier domain, the low-frequency components generally carry greater weight than the high-frequency components, and there is a radial correlation between low frequencies and high frequencies in the Fourier spectrum. High-frequency components with high energy tend to cluster along certain angles emanating from the central zero frequency, giving the spectrum an overall starburst pattern with a bright center. Therefore, for static targets, adaptive sampling strategies that predict important high-frequency content from low-frequency measurements effectively improve reconstruction quality.

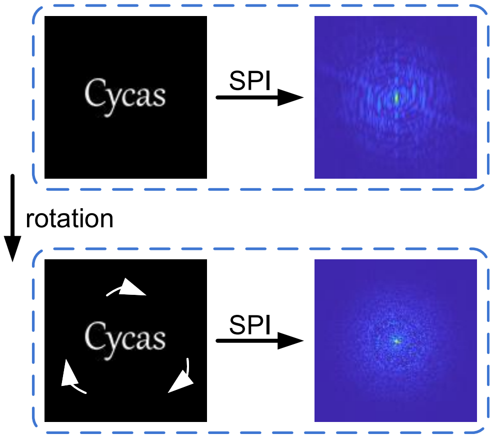

Supplementary Fig. 4: The effect of rotation on the Fourier spectrum in the SPI perspective.

However, if the target undergoes an arbitrary rotation during the sampling interval, its Fourier spectrum will rotate accordingly, causing distortion and loss of radial correlation in the Fourier-SPI view, as illustrated in Supplementary Fig.4. Hence, when the target exhibits rotational motion, especially

random rotations without any prior information, the appropriate sampling strategy is a circular diffusion scheme that starts from the central zero frequency and proceeds uniformly from low to high frequencies in all directions.

Special sampling strategy of the extend-FOV imaging of a boundary-motion target

In the extend-FOV imaging of a boundary-motion target, the situation is somewhat different. Since each measurement frame simultaneously captures both target localization and image information, when we filter trajectories based on the motion state, some of the imaging measurement values are inevitably discarded. Moreover, this discard is entirely based on the target's motion, so we cannot predict the exact information that will be discarded. The Fourier acquisition method is a compressive sampling technique in which each sample is not equivalent. Given that most of the information in natural scenes is concentrated in the low-frequency region and that low-frequency data carries much more weight than high-frequency data, using the traditional strategy of sequentially acquiring the Fourier spectrum from low to high frequencies may result in the loss of critical low-frequency information due to random discards, thereby severely affecting the quality of the reconstructed image.

To ensure that enough key low-frequency information is obtained, we repeatedly sample using the patterns corresponding to the central 10% low-frequency region of the Fourier spectrum, foregoing the acquisition of higher-frequency information.

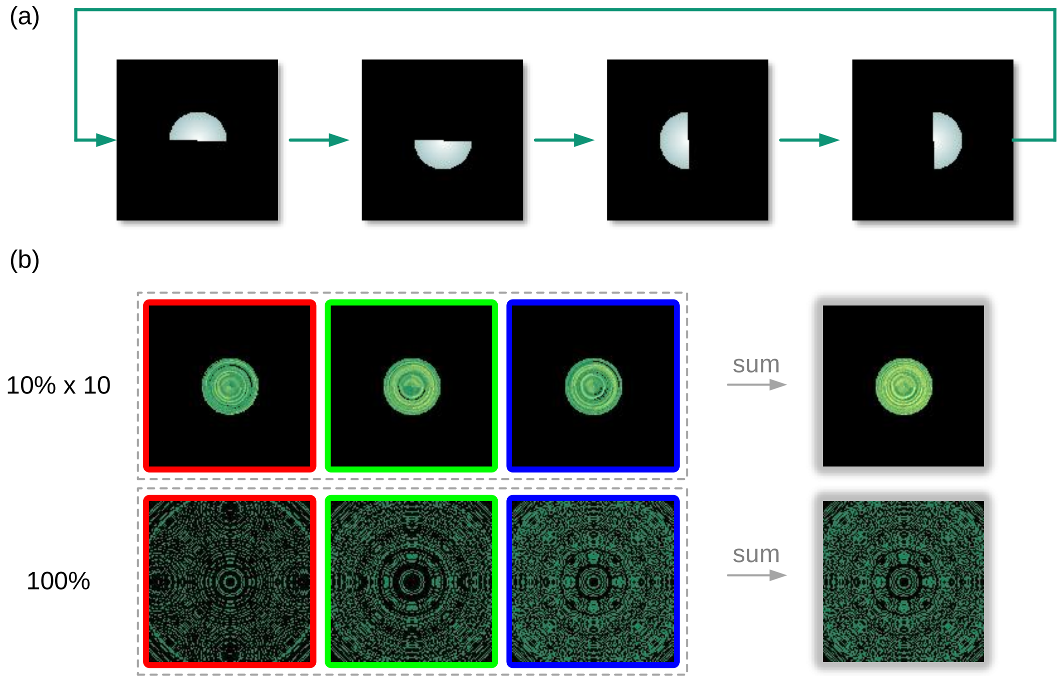

Supplementary Fig. 5: Sampling Strategy and Spectral Coverage in the extend-FOV imaging of a boundary-motion target. (a) Sampling strategy used in extended FOV imaging: focus on the central 10% of the Fourier spectrum and cyclically acquire the upper, lower, left, and right semi sectors to improve spectral coverage.(b) Spectral coverage results from the real experiment reported in the main text, showing the coverage for each of the RGB channels and the overall spectrum.

In our experiments, based on the conjugate symmetry of the center of the Fourier spectrum and the fact that symmetric sampling in a rotating system is not equivalent, we sequentially acquire the upper, lower, left, and right parts of the Fourier spectrum, as shown in the Supplementary Fig.5(a). Using real experimental results of the extend-FOV imaging of a boundary-motion target, we compared our optimized repeated sampling strategy for the central 10% low-frequency region with the traditional full sampling strategy, showing the spectral coverage for the RGB channels and the entirety in Fig.5(b).

Our method increases the sampling density in the low-frequency region and reduces the loss of critical information, so that enhances the system's reliability.

Supplementary Note 3: Experimental platform device models

The target is mounted on a two-axis combined translation stage (CXPF100-80175, TBRF75L, Zolix) to simulate composite translational and rotational motion. Illumination is provided by a 4.6 W high-power LED source (SOLIS-3C, THORLABS). The target is imaged through a zoom lens (MVL5M23, THORLABS) and then relayed by a relay lens (AC254-030-A-ML, THORLABS) to project an appropriately sized image onto the central region of a digital micromirror device (DMD; DLP7000, Texas Instruments). On the +1 side of the DMD, the modulated signal is split by two dichroic mirrors (DMR-490LP and DMR-605LP, LBTEK), at cut-off wavelengths of 490 nm and 605 nm, into three spectral channels which correspond to the RGB channels. These optical signals were then collected by lens (AC4704-A, LBTEK) and detected by three silicon-amplified photodetectors (PDA100A2, THORLABS) operating as single-pixel detectors, enabling multichannel sensing of the target. The voltage signals from the detectors were recorded by a data acquisition card (USB-6366, National Instruments) and processed on a workstation (CPU: Ryzen 9 9950X; RAM: 96 GB).

Supplementary Note 4: Pattern setup

The patterns are generated by the computer, consisting of a 768×768 pixel Fourier localization patterns with spatial frequencies of 1/2M and 1/2N (M = 768, N = 768), and a 128×128 pixel Fourier or Hadamard imaging patterns with an upsampling ratio of 6 (a 6 × 6 block of physical pixels on the DMD is binned to emulate a single virtual pixel). These are combined into a single frame of measurement, simultaneously capturing the target's motion state and image information. As described in Supplementary Note 1, Fourier basis functions with spatial frequencies of 1/2M and 1/2N provide the best localization accuracy within the SPI localization system. Both the Fourier localization patterns and the Fourier imaging patterns are generated using a three-step phase-shifting method and are binarized using the Sierra-Lite method with a serpentine diffusion path to achieve better locating accuracy and image quality. The Hadamard pattern is generated using a differential measurement approach. In summary, each measurement frame in Fourier-SPI comprises 9 patterns (3 for localizing the absolute coordinate along y, 3 for localizing the absolute coordinate along x, and 3 for imaging); in Hadamard-SPI, each frame comprises 8 patterns (3 for localizing the absolute coordinate along y, 3 for localizing the absolute coordinate along x, and 2 for imaging).

In Fourier-SPI, using the sampling strategy shown in Supplementary Note 2, full sampling of the target requires 49,152 imaging patterns. For each Fourier coefficient, 6 corresponding localization patterns are needed, bringing the total number of patterns to 147,456. In Hadamard-SPI, complete sampling requires 32,768 imaging patterns. Similarly, calculating localization patterns, the total number of patterns required is 131,072. In our real experiments, we use a 50% sampling ratio and set the DMD to the lowest feasible flipping rate, for example 150 Hz. Under this setting, a single Fourier-SPI acquisition requires 492 s, thereby allowing the target ample time to undergo substantial translational and rotational motion. If the DMD operates at its maximum flipping rate (22000 Hz), a single acquisition takes only 3.35 s.

Advanced setups

Additional options exist to further improve temporal resolution; however, our aim is to present an overall framework, and the main text does not employ such advanced configurations.

Under the assumption that the object is approximately stationary within each frame, we exploit the property that the three patterns produced by three-step phase shifting sum to an all-1 pattern. A single all-1 pattern is used to replace one pattern in each of the three phase-shift sets within each measurement frame, which reduces the per-frame measurement by 2 patterns. A more aggressive option is applicable when the target remains within the field of view throughout the acquisition and its total

intensity is assumed constant over all measurements. In this case, the first all-ones pattern in the three-step phase-shifting for the zero-frequency Fourier coefficient can be used as a reference, thereby reducing the number of patterns per measurement frame by three.

Moreover, our method provides a tunable allocation of resources between localization temporal resolution and imaging temporal resolution. In the current configuration, each frame acquires only one imaging coefficient to maintain a high temporal resolution of localization. If multiple imaging coefficients are acquired within a frame, the per-frame measurement time increases, thereby reducing the localization temporal resolution, while the total number of frames required to complete a reconstruction decreases, shortening the overall imaging time. Based on this trade-off, the number of imaging coefficients per frame and the set of basis patterns can be flexibly configured according to the target motion speed and the scene requirements, enabling the system to achieve an optimal balance under different operating conditions.

Supplementary Note 5: Numerical simulation of MC3-SPI

We conducted numerical simulations to validate the effectiveness of MC3-SPI. Our target was a colored bird composed of tangram pieces, with a size of 280×280 pixels, which occupies approximately 1/9 of the FOV, which is a typical multicentroid target in the wavelength channel. In the simulation, at virtual time t, the target's absolute position $\big(x(t), y(t)\big)$ and rotation angle $\theta(t)$ are given by:

$$\begin{aligned} x(t) &= \text{round}((N-K-2)cos(2\pi vt+1.2)/2/(1+vt)+(M-K)/2), \\ y(t) &= \text{round}((M-K-2)cos(2\pi vt)/2/(1+vt)+(M-K)/2), \\ \theta(t) &= 0.93*360*((1+sin(2\pi\omega t))/2+(1+10cos(2\pi*0.513t+1.02))/11 \\ &\quad +(2+cos(2\pi*0.232t+0.52))/3)/3, \end{aligned} \tag{18}$$

where, $t=(n-1)\Delta t$, with n indexing the current frame, corresponding to the n-th SPI pattern. And Δt denotes the minimum temporal resolution of the system. In this simulation, the DMD operates at 20 kHz, which corresponds to a temporal resolution of $1/20\,000\,\text{s}$. M and N denote the pixel dimensions of the SPI field of view, with both set to 768. K represents the target pixel size in the SPI perspective and is set to 280. The parameter v characterizes the translational motion of the target and is fixed at 0.618 in this simulation, while ω represents the rotational motion of the target and is fixed at 3.14.

To rigorously assess the efficacy of our approach, we prescribe a motion trajectory of adequate complexity, deliberately increasing the complexity of the rotational component. Note that in the numerical simulations both translation and rotation are defined with respect to the geometric center of the target image, whereas our method estimates motion relative to the geometric centroid of the three-channel centroids. Because these two centers do not coincide, the translational trajectory recovered by our method differs from that in Eq.18. This discrepancy is a purely referential effect arising from the choice of origin and does not affect the quality of the reconstructed images.

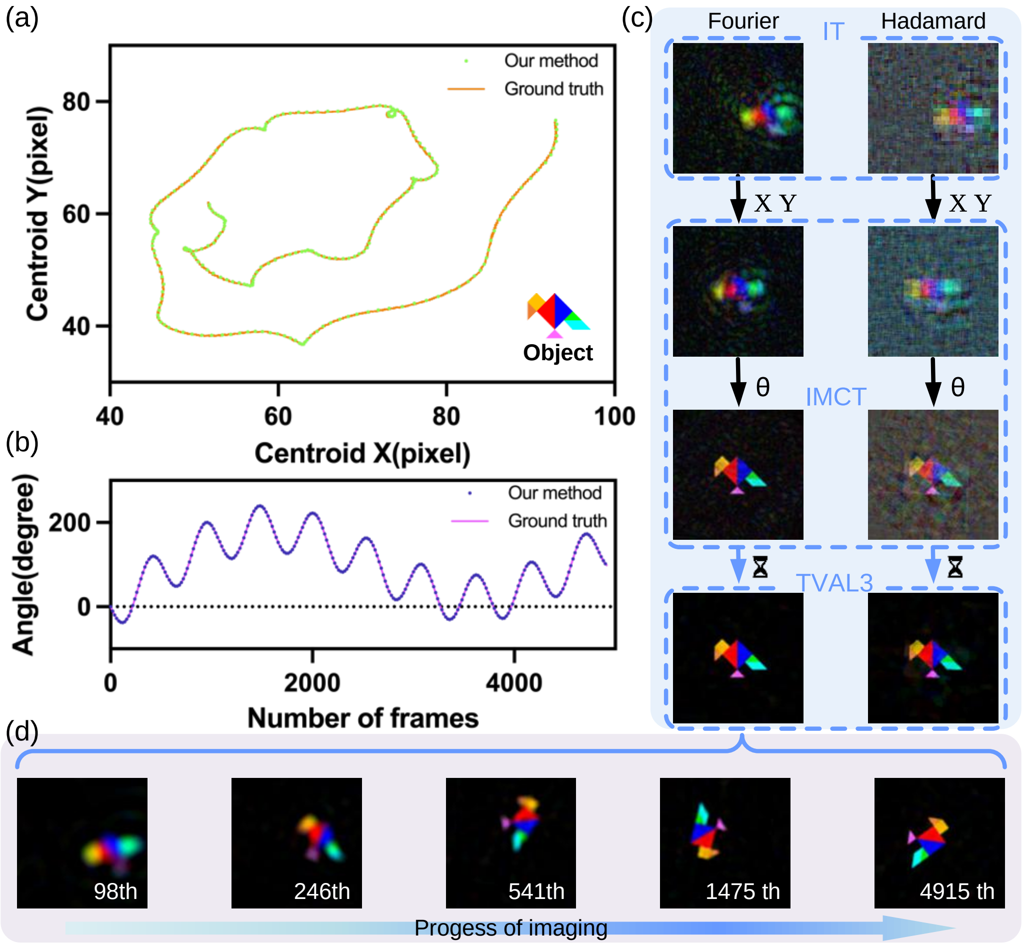

Supplementary Fig. 6: The simulation results for tracking and imaging the colored tangram bird are presented. (a) The measured translational trajectory of the target, compared with the ground truth. (b) The measured rotation angles of the target, compared with the ground truth. (c) The IMCT reconstructions obtained by both the Fourier and Hadamard methods under three scenarios: no motion compensation, translational compensation only, and simultaneous translational and rotational compensation. And the TVAL3 method can achieve superior reconstruction results, but at the expense of additional computation time. (d) Five slices from the sampling process using the Fourier-TVAL3 method are presented, which reproduce the target's motion and imaging process.

The settings for the localization and imaging patterns were identical to those used in the real experiments: the localization pattern was 728×728 pixels, and the imaging pattern was 128×128 pixels with an upsampling ratio of 6 to match the size of the localization pattern. Fourier patterns were implemented with a three-step phase-shifting method using the Serria-Lite dithering method along a serpentine error diffusion path, while Hadamard patterns were acquired using positive-negative differential sampling. The sampling rate was set at 30%. The numerical simulation results are shown in Supplementary Fig. 6, which can be regarded as ideal experimental results with no noise, no optical distortions, and the target's motion plane strictly perpendicular to the optical axis.

As shown in the trajectory plots, our method's tracking of the target's compound motion highly coincides with the ground truth, which fully validates its accuracy in capturing the two-dimensional composite motion states of multicentroid targets. In the reconstruction results, after sequentially correcting translation and rotation, we recovered a clear image of the target, demonstrating the effectiveness of our compensation method for imaging moving targets. Moreover, in the IMCT method, the imaging results achieved using the Fourier method are significantly superior to those obtained using the Hadamard method; in TVAL3, the Fourier method also outperforms the Hadamard method in terms of edge detail. These findings are further corroborated by experimental results, confirming the advantages of the Fourier method in handling complex moving targets.

Based on the complete two-dimensional motion information of the target and the scanning results of its Fourier spectrum, we can reconstruct the entire imaging process. We selected five nodes and reproduced the target's motion state and the corresponding imaging results at those moments.It is evident that the reconstructed target image becomes progressively clearer as the sampling rate increases, and that the motion of the target contains both translational and rotational components. Furthermore, we observed that at the 541st measurement, corresponding to a sampling rate of 5%, the approximate outline of the target could be obtained, enabling rapid target recognition.

Supplementary Note 6: Robustness analysis of MC3-SPI

We conducted a robustness analysis of our approach by incorporating Gaussian noise into the numerical simulation settings established in Supplementary Note 5. We employ MATLAB's awgn() function to inject additive white Gaussian noise into the signal, setting the "signalpower" option to "measured" so that the routine first computes the signal level and then determines the required noise level.

We use the signal-to-noise ratio (SNR) to represent the noise level, as given by the following equation:

$$\mathrm{SNR_{dB}} = 10\log_{10}\left(\frac{P_{\mathrm{signal}}}{P_{\mathrm{noise}}}\right) \ or\ 20\log_{10}\left(\frac{A_{\mathrm{signal}}}{A_{\mathrm{noise}}}\right), \tag{19}$$

where, P is the power and A is the root mean square (RMS) amplitude.

We use the root mean square error (RMSE) to quantify the accuracy of attitude sensing, as given by the following equation:

$$\mathrm{RMSE}_{xy\ or\ \theta} = \sqrt{\frac{1}{n}\sum_{i=1}^{n}\left((x_i-\tilde{x}_i)^2+(y_i-\tilde{y}_i)^2\right)} \ or\ \sqrt{\frac{1}{n}\sum_{i=1}^{n}\left(\theta_i-\tilde{\theta}_i\right)^2}, \tag{20}$$

where x_{i}, y_{i} and θ_{i} are the true value, x_{i}, y_{i} and θ_{i} are the predicted or estimated value, and n is the number of samples.

For two images, x and y, the structural similarity (SSIM) is calculated by the following equation:

$$\mathrm{SSIM}(x,y) = \frac{(2\mu_x\mu_y + C_1)(2\sigma_{xy} + C_2)}{(\mu_x^2 + \mu_y^2 + C_1)(\sigma_x^2 + \sigma_y^2 + C_2)}, \tag{21}$$

where, μ_x and μ_y are averages of the two compared images, σ_x^2 and σ_y^2 are the variances of the images, σ_{xy} is the covariance, and C1 and C2 are two variables calculated from the dynamic range of the images.

For two images, x and y of sizes [M, N], the peak signal-to-noise ratio (PSNR) is calculated by the following equation:

$$\mathrm{PSNR}(x,y) = 10\log_{10}\left(\frac{\mathrm{MAX}_x^2}{\mathrm{MSE}}\right) = 10\log_{10}\left(\frac{\mathrm{MAX}_x^2}{\frac{1}{MN}\sum_{i=1}^{M}\sum_{j=1}^{N}\left(x(i,j)-y(i,j)\right)^2}\right), \tag{22}$$

where, MAX_x is the maximum possible pixel value of the image (for an 8 bit image, MAX_x =255), and MSE is the mean squared error between the reference and reconstructed images.

We first conduct a numerical simulation of intrinsic noise / dark noise of the detector. In supplementary Fig. 7(a) and Fig. 7(b), the results indicate that even with an SNR of 40 dB, sufficient localization accuracy is maintained. Notably, higher accuracy is required for the rotation angle in motion compensation. The estimation of the rotation angle is more severely affected by noise, resulting in higher RMSE values and larger fluctuations (error bars). Therefore, the overall robustness of the system noise is constrained by the accuracy of the angle estimation.

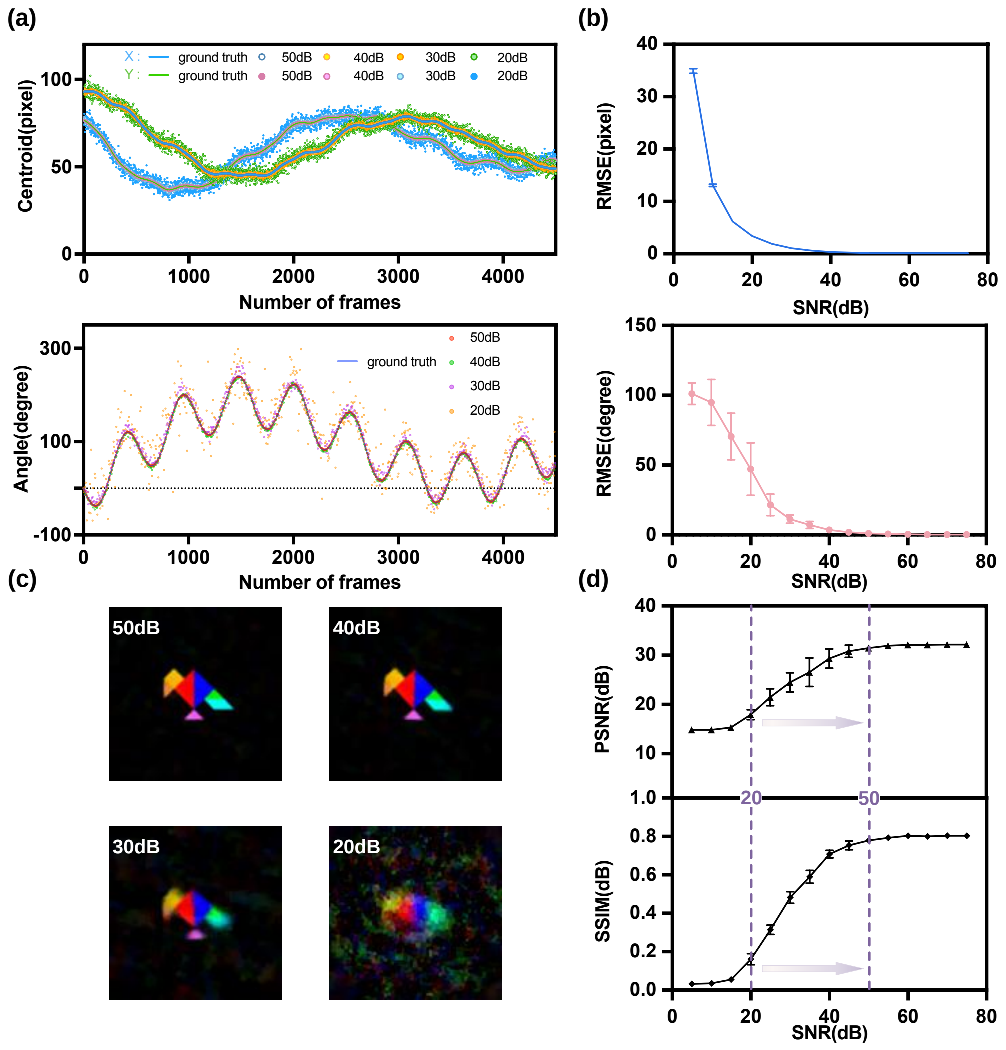

Supplementary Fig. 7: Numerical simulation results under different Gaussian noise levels (intrinsic noise / dark noise). (a) Tracking results of the target's composite motion at signal-to-noise ratios of 50 dB, 40 dB, 30 dB, and 20 dB. (b) RMSE of the tracking results under various noise levels. (c) Image reconstruction results of the Fourier method at signal-to-noise ratios of 50 dB, 40 dB, 30 dB, and 20 dB. (d) PSNR and SSIM of the Fourier method's image reconstruction results under different noise levels.

In Supplementary Figs. 7 (c) and (d), as the SNR decreases from 50 dB to 40 dB, noise primarily affects the acquisition of image information. Due to the compressive nature of the Fourier method, which provides inherent robustness under low sampling conditions, the image quality only degrades slightly. However, as the SNR drops from 40 dB to 30 dB, angle estimation begins to fail, resulting in noticeable motion blur in the rotational direction and a significant decline in the quality of the reconstructed image. With a further reduction of the SNR to 20 dB, the reconstructed image becomes indiscernible, and our method completely fails. Especially, as the SNR decreases, the reconstructed image first exhibits motion blur in the rotational direction, further emphasizing the critical importance of accurate angle estimation in our approach. Therefore, our method is capable of faithfully reconstructing the target image at a noise level of approximately 40 dB.

In particular, the silicon-amplified photodetectors (PDA100A2, Thorlabs) used in our setup deliver a RMS dark output of 229μV at the gain mode of 40dB setting, while the signal level measured during the experiments is about 0.5V. Therefore, the SNR of the photodetector for a single readout is approximately 66.78dB ($\Delta \mathrm{SNR}_{\mathrm{dB}} = 20 \log_{10} \frac{RO}{DCR}$, $RO = 0.5\mathrm{V}$ $DCR = 229 \times 10^{-6}\mathrm{V}$).

Beside, the data acquisition card (USB-6366, National Instruments) can record 2×10^6 samples per

second. The DMD (DLP7000, Texas Instruments) has a maximum flipping rate of 22 kHz, which implies that each pattern can be sampled at least 90 times. Because the signal component is coherent whereas the noise is not, repeated measurements cause the signal to add linearly as N while the noise grows only as $\sqrt{N}$. The signal accumulates $\sqrt{N}$ times faster than the noise. Ideally, this would provide an improvement in the SNR of approximately 19.50dB ($\Delta \mathrm{SNR}_{\mathrm{dB}} = 20 \log_{10} \sqrt{N},\ N = 90$).

After all, the resulting SNR for a single pattern acquisition is approximately 86.30dB. This SNR level enables the reconstruction of high-quality images of targets undergoing compound motion.

In optical imaging systems, another common noise source arises from ambient background illumination. It is worth noting that SPI senses the image holistically, with each measurement records the sum of the signals from numerous pixels across the field of view. In Fourier-SPI, a single readout corresponds to approximately half of the total optical power of the scene. Due to this integrative acquisition principle, SPI is inherently highly robust to background noise[6]. To quantify this robustness, we analyze the system response to background noise on top of its intrinsic noise floor of 86.30 dB. The result is shown in Supplementary Fig. 8.

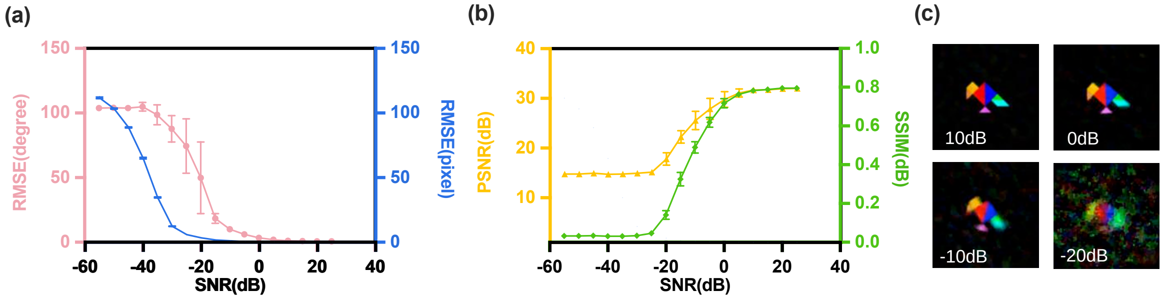

Supplementary Fig. 8: Numerical simulation results under different Gaussian noise levels (background noise). (a) RMSE of the tracking results under various noise levels. (b) PSNR and SSIM of the Fourier method's image reconstruction results under different noise levels. (c) Image reconstruction results of the Fourier method at signal-to-noise ratios of 10 dB, 0 dB, −10 dB, and −20 dB.

As shown in Supplementary Figs. 8 (a) and (b), the SPI exhibits strong robustness to background noise. Even at a background noise level of −10 dB, it can still reconstruct an approximate image of the target. In Supplementary Figs. 8 (c), a clear image can be reconstructed when the background noise level is 0 dB. Consequently, the proposed method with our system can faithfully reconstruct the target image even at ambient noise levels of ~ 0 dB.

Due to the Fourier localization method exhibits excellent robustness, We provide a brief discussion of the robustness of the geometric moment (GM) localization method. Numerical simulations illustrate the results of the GM localization method under SNRs of 70 dB, 80 dB, and 90 dB, as shown in the Supplementary Fig. 9.

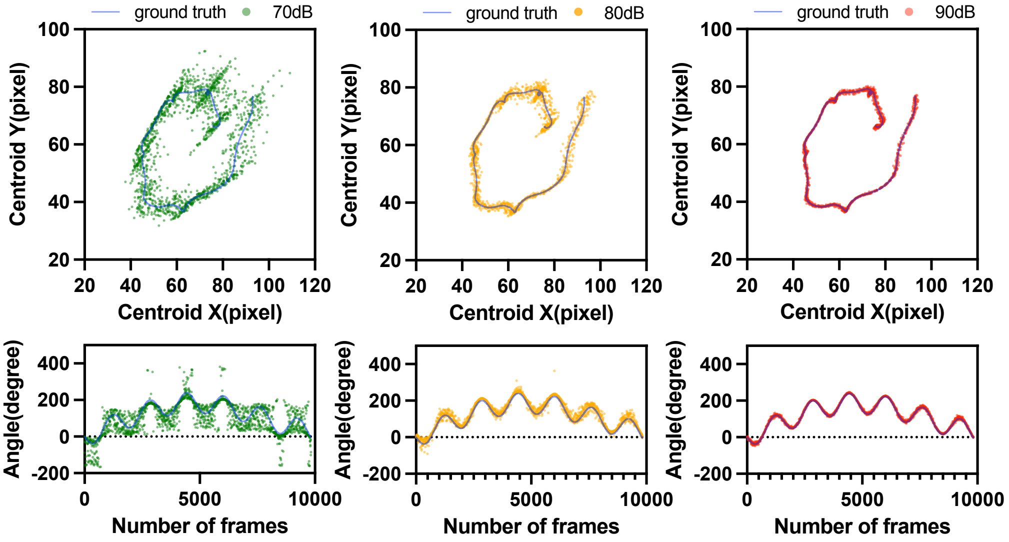

Supplementary Fig. 9: Numerical simulation results of the GM localization method under different Gaussian noise levels (intrinsic noise / dark noise). The GM method requires an SNR of at least 90 dB to achieve reasonably accurate localization; when the SNR drops to 70 dB, localization accuracy deteriorates severely, and angle acquisition fails completely.

We can clearly see that the robustness of the GM method is far inferior to that of the Fourier method, as it completely fails at an SNR of 70 dB. Specifically, at 40 dB, the RMSE of GM localization (the geometric center of the three-channel centroids) is approximately 200 pixels, whereas the Fourier localization method achieves about 0.36 pixels, three orders of magnitude better. With ambient noise neglected and DMD at its maximum flip rate, each measurement attains an SNR of ~ 86.3 dB. The GM localization error is ~ 0.81 pixels with the angular estimation error being $\sim 11.2°$. Under current experimental conditions, implementing our method requires lowering the DMD flipping rate to obtain a higher SNR, which comes at the cost of a reduced temporal resolution.

The GM method is based on the definition of the centroid and directly yields the absolute coordinate of the target centroid[7]; however, its lack of complementary measurements makes it highly sensitive to noise.

Supplementary Note 7: Performance analysis of Fourier and Hadamard basis patterns with motion-compensation

For moving targets in SPI, applying a reverse motion to the modulation matrix is an effective strategy to mitigate motion blur caused by target movement. However, this process is not always lossless in SPI reconstruction. Therefore, selecting an appropriate modulation pattern for different motion modes can significantly enhance both the efficiency and quality of target image reconstruction. Our approach requires compensation for both translation and rotation. Both simulation and experimental results show that, regardless of the motion mode or target shape, the reconstruction results obtained with the Fourier method are consistently and markedly superior to those from the Hadamard method. Specifically, with the IMCT method, the Fourier approach yields images with a noticeably cleaner background; while with the TVAL3 algorithm, it provides richer image details.

In conventional SPI, both the Fourier and Hadamard basis patterns offer high reconstruction efficiency due to their orthogonal completeness and sparsity in the transform domain[8]. Therefore, we focused our analysis on the orthogonality of these basis patterns after undergoing translation or rotation. To quantitatively assess this orthogonality, for a set of orthogonal patterns $\{P_1, P_2, ..., P_n\}$, we employ the normalized Frobenius inner product[9] (matrix inner product) between the compensated basis pattern

P_i' and the remaining basis patterns $P_{j\ (j\neq i)}$, as shown in the following equation:

$$C_i = \frac{MN}{n-1}\sum_{j\neq i}\frac{\left|\,\langle P_i', P_j\rangle_F\,\right|}{\|P_i'\|_F\,\|P_j\|_F},\qquad 0\le C_i\le MN. \tag{23}$$

where, Frobenius inner product $\langle A, B\rangle_F = \mathrm{tr}\big(A^{\mathrm{H}}B\big)$, Frobenius norm $\|A\|_F = \sqrt{\langle A, A\rangle_F}$. n denotes the number of complete orthogonal bases, (M, N) denotes the size of the bases. A smaller C_i means the patterns are closer to orthogonal.

The Eq.23 describes the correlation between the compensated basis pattern and the remaining basis patterns. We multiply the mean normalized Frobenius inner product by the size of the basis patterns to associate the result with the number of pixels. The metric C_i is termed the scaled mean normalized Frobenius inner-product magnitude (sMNFI).

In compressed sensing, mutual coherence quantifies how similar the column vectors of a sensing matrix (or dictionary) are[10, 11]. Given $A = [a_1, \ldots, a_n]$ with $\|a_i\|_2 = 1$ for all i, it is defined as

$$\mu(A) = \max_{i\neq j}\,|\langle a_i, a_j\rangle|,\qquad 0\le\mu\le 1. \tag{24}$$

A smaller μ means that the columns are closer to orthogonal and sparse recovery is more robust.

Eq.23 and Eq.24 essentially describe the same quantity, differing only in emphasis. In compressed sensing, the maximum mutual coherence is employed to characterize the worst case and ensure the stability of sparse recovery. In our setting, we examine the correlation between a motion-compensated basis pattern and the remainder of the orthogonal basis pattern set to quantify the orthogonality loss introduced by motion-compensation, and therefore we use the mean mutual coherence to capture the overall average similarity.

In SPI, we aim for each basis pattern to be uncorrelated with the others to avoid redundant sensing of target information, thereby enhancing the measurement efficiency. The smaller the value of this inner product, the better the orthogonality between the compensated basis pattern and the remaining ones. This indicates that the loss of orthogonality due to the compensation operation is minimized, leading to an improved theoretical reconstruction performance.

We sorted the basis patterns according to the SPI sampling strategy and selected the top 5%, 15%, 30%, 50% of the patterns. From each set of basis patterns, 800 patterns are randomly selected. For each selected pattern, random motion compensation is applied and the mean inner product is computed, with each pattern repeated 100 times. For each basis pattern, an arbitrary translation or rotation or translation and rotation is applied, after which its average Frobenius inner product with the remaining patterns is calculated, as shown in Eq.23. This procedure was repeated 100 times per basis pattern. The statistical results are shown in the Supplementary Fig.10. Compared with the Hadamard basis, the average Frobenius inner product distribution for the Fourier basis is more concentrated near zero, indicating that after translation and rotation operations, the Fourier basis better preserves orthogonality; this advantage is especially pronounced under low sampling rate conditions.

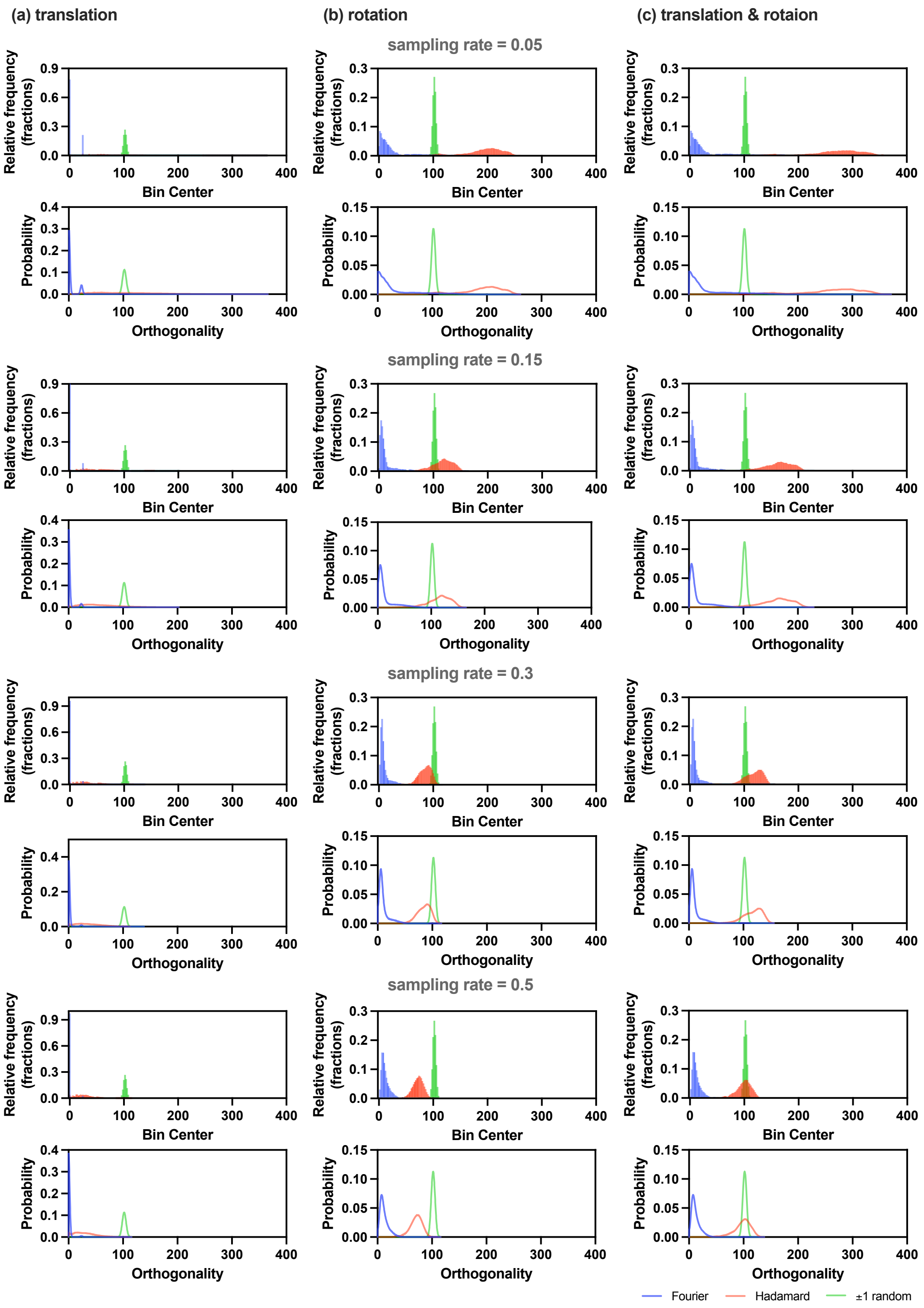

Supplementary Fig. 10: Orthogonality analysis of basis patterns at different sampling rates after (a) translation or (b) rotation or (c) translation and rotation is described using the mean normalized Frobenius inner product. The closer the data distribution is to zero, the better the orthogonality of the basis patterns after motion compensation. Basis patterns of size 128×128 are used, with sampling ratios set to 5%, 15%, 30%, and 50%. In each subplot, the up panel shows the statistical results of the data, and the down panel displays the probability density function estimated with a normal kernel function and a bandwidth of 2. Random ± 1 patterns are used as a control to benchmark the orthogonality level.

To further substantiate our conclusions, we evaluated the orthogonality of the complete sets of Fourier basis patterns and Hadamard basis patterns under motion compensation. For each basis pattern, we

applied random motion-compensation transforms of various types and computed the mean normalized Frobenius inner product with the remaining basis patterns. This procedure was repeated 100 times. The results in Supplementary Fig.11 indicate that the orthogonality of the Fourier basis patterns is more robust to motion compensation than that of the Hadamard basis patterns. Consequently, when performing motion-compensated imaging of moving targets, Fourier-SPI supports effective reconstruction over a wider range of sampling rates, whereas Hadamar-SPI requires higher sampling rates to maintain ensemble orthogonality and preserve image quality.

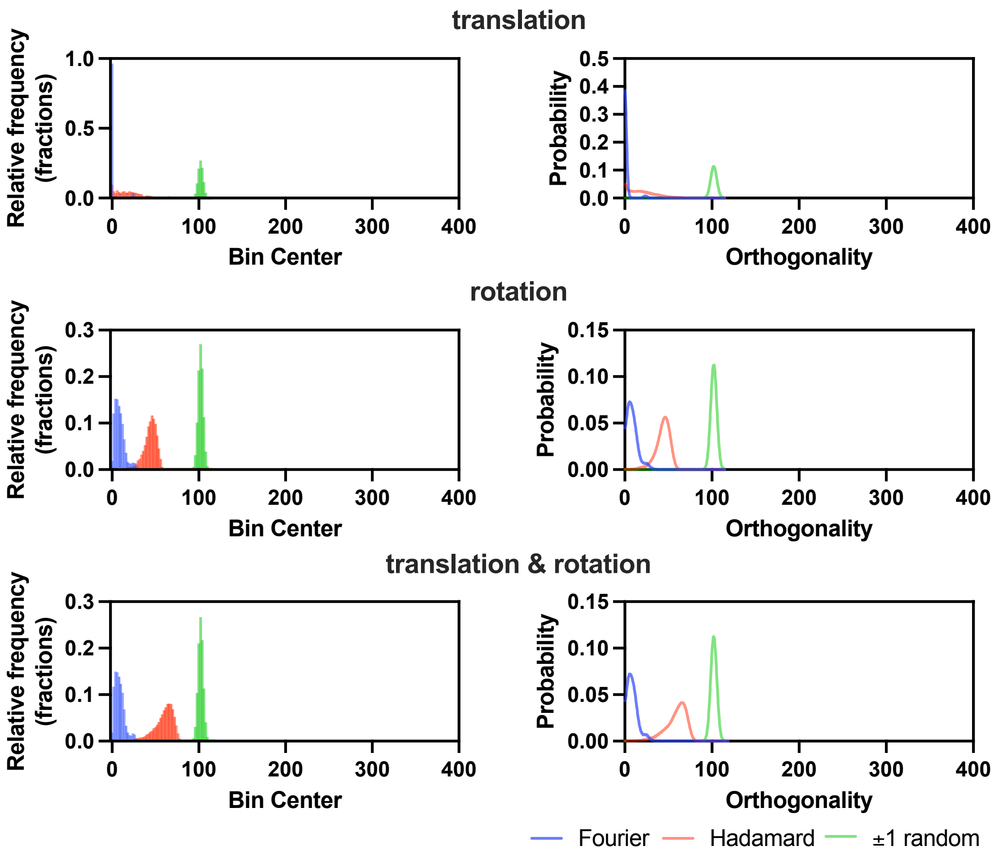

Supplementary Fig. 11: Orthogonality analysis of the complete basis pattern sets after translation or rotation or translation and rotation is described using the mean normalized Frobenius inner product. The closer the data distribution is to zero, the better the orthogonality of the basis patterns after motion compensation. In plot, the left panel shows the statistical results of the data, and the right panel displays the probability density function estimated with a normal kernel function and a bandwidth of 2. Random ± 1 patterns are used as a control to benchmark the orthogonality level.

This difference arises from the fact that the discrete Fourier transform is derived from the discretization of the continuous Fourier transform, whereas the Hadamard basis is inherently discrete. The discrete Fourier basis can be understood as the result of discarding a portion of the continuous Fourier basis due to resolution limitations. In the process of rotational compensation, mathematically, the rotated continuous Fourier basis corresponds to the discarded continuous Fourier basis, so the rotation does not affect the orthogonality of the Fourier basis. The mathematical expression for a Fourier basis pattern rotated counterclockwise by an angle θ about the origin is given by:

$$\begin{aligned} P(x', y' \mid f_x, f_y) &= e^{-2\pi j\left(f_x x' + f_y y'\right)} \\ &= e^{-2\pi j[f_x(\cos\theta\cdot x - \sin\theta\cdot y) + f_y(\cos\theta\cdot y + \sin\theta\cdot x)]} \\ &= e^{-2\pi j[(\cos\theta\cdot f_x + \sin\theta\cdot f_y)x + (\cos\theta\cdot f_y - \sin\theta\cdot f_x)y]} \\ &= P(x, y \mid f'_x, f'_y). \end{aligned} \tag{25}$$

From the above equation, it is evident that rotating the Fourier basis in the spatial domain is equivalent to a reverse rotation in the frequency domain, ensuring strict mathematical orthogonality before and after the transformation. For translation compensation, the periodic nature of the Fourier basis means that translation essentially affects only the phase. Mathematically, this is equivalent to multiplying the original Fourier basis by a single coefficient, and thus does not impact the orthogonality of the measurement patterns. The mathematical expression for a Fourier basis pattern after a cyclic translation $(\mathrm{d}x, \mathrm{d}y)$ is given by the following:

$$
\begin{aligned}
P(x', y' \mid f_x, f_y) &= e^{-2\pi j(f_x x' + f_y y')} \\
&= e^{-2\pi j[f_x(x-dx)+f_y(y-dy)]} \\
&= e^{-2\pi j(f_x x + f_y y - f_x dx - f_y dy)} \\
&= e^{-2\pi j(f_x dx + f_y dy)} \cdot P(x, y \mid f_x, f_y)
\end{aligned}
\tag{26}
$$

Due to the decomposability of two-dimensional motion, the properties of the above two equations extend to any planar motion. Consequently, we observe that mathematically, a complete set of continuous Fourier bases forms a linear space that is closed under planar motions. In an SPI system, continuous Fourier basis patterns retain orthogonality under arbitrary translations and rotations. However, resolution limits and interpolation cause a partial loss of orthogonality in measurement patterns after motion compensation.

In Hadamard-SPI, the 2^n-order Hadamard matrix is recursively constructed from the first-order Hadamard matrix. Consequently, the resulting Hadamard basis patterns, due to their inherent discreteness and block-like pattern characteristics, experience a significant degradation in orthogonality under both translation and rotation.

On the other hand, the discrete Cartesian coordinate system cannot perfectly describe rotational motion. In practical applications, when rotating the basis patterns, interpolation methods (bilinear and bicubic, as employed in this paper) must be employed, as illustrated in the Supplementary Fig.12. This results in sharply defined Hadamard patterns becoming blurred at the interfaces between +1 and −1 after rotation, leading to a loss of structural features[12]. In contrast, the continuous grayscale properties of the Fourier basis allow it to better preserve its fringe characteristics during interpolation-based rotation. The degradation of the structure of the basis pattern adversely affects the capture of the target morphology, which subsequently leads to the loss of fine details in the reconstructed image.

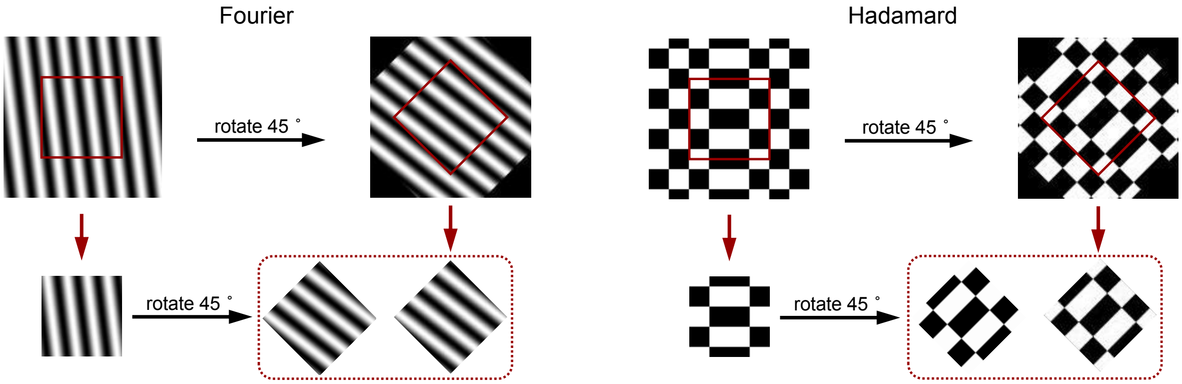

Supplementary Fig. 12: A morphological comparison of the Fourier and Hadamard basis patterns before and after rotation. We selected the central region of the pattern because it is most relevant to the target image. In the inset, the left panel shows the result of direct rotation (effectively lossless), while the right panel shows the result of the interpolated rotation in IMCT.

In summary, the Fourier basis patterns maintain better orthogonality during motion compensation, and the interpolation used in rotational compensation does not lead to excessive morphological degradation. These characteristics ensure both the efficiency of SPI in dealing with targets that exhibit composite translational and rotational motion and the preservation of fine target details, thereby achieving

superior imaging results. Most importantly, the complete continuous Fourier basis space is closed under translations and rotations, making Fourier patterns the optimal choice for imaging targets that undergo both translational and rotational motion in SPI.

Supplementary Note 8: Analysis of target image reconstruction results under different sampling rates for IMCFT

Motion compensation, especially rotational compensation, is challenging because the discrete Cartesian coordinate system cannot perfectly represent rotation. This results in varying degrees of degradation in the basis patterns before and after rotation within the SPI system. The continuous periodic grayscale stripe features of Fourier patterns are well suited for both translational and rotational compensation (Supplementary Note 7). However, Fourier basis patterns still experience some weakening of their orthogonality after rotation, which can impact the reconstruction efficiency of IMCFT.

Thus, we present IMCFT reconstruction results at different sampling rates with our real experimental data to visually demonstrate the imaging efficiency of the Fourier method. We particularly focus on reconstructions with sampling rates below 10%, as shown in Supplementary Fig.13.

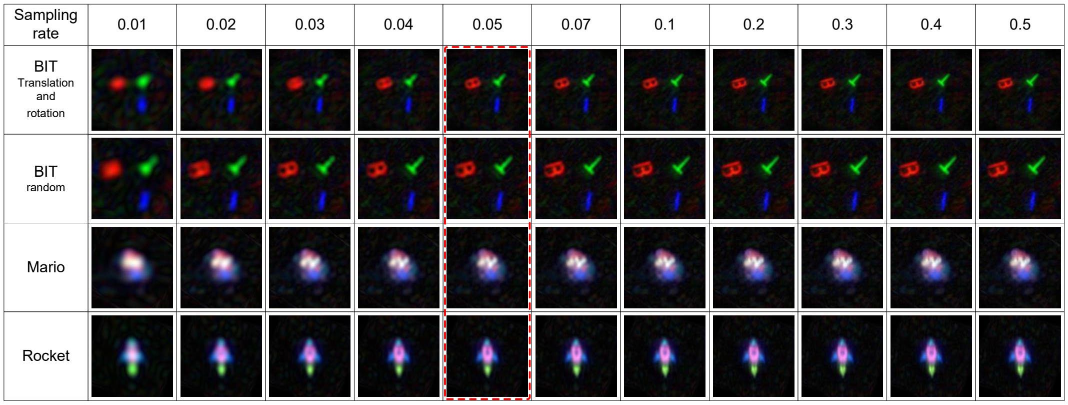

Supplementary Fig. 13: IMCFT reconstruction results under different sampling rates.

To further quantify the efficiency of IMCFT reconstruction, we selected two experimental data sets (Mario and Rocket) and computed the SSIM and PSNR metrics for the IMCFT reconstruction results at various sampling rates, using the results obtained with the TVAL3 algorithm at a sampling rate 50% as the reference. We manually selected the region containing the target to mitigate the influence of background noise, introduced by the rotation of basis patterns, on global image quality metrics such as PSNR and SSIM. Detailed results are provided in the Supplementary Fig.14.

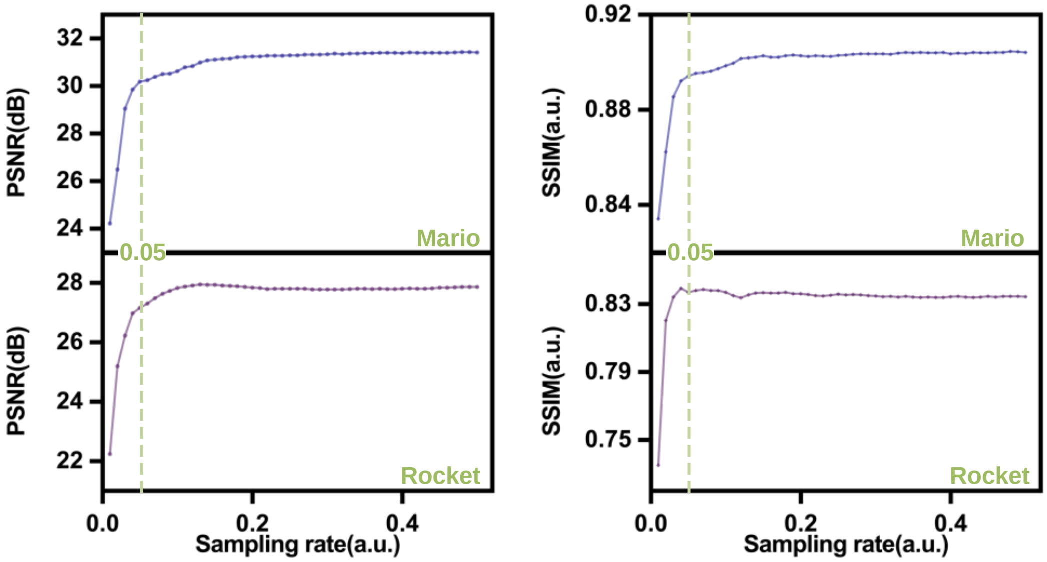

Supplementary Fig. 14: PSNR and SSIM for Mario toy and rocket toy results at different sampling rates. The results reconstructed using the TVAL3 algorithm at a sampling rate 50% are used as the reference.

Similarly to traditional Fourier-SPI, using a sampling strategy that proceeds sequentially from low to high frequencies, the image quality reconstructed by the IMCFT method converges rapidly and improves significantly. We can observe that even at a mere 1% sampling rate, the scene can be roughly identified; however, when the sampling rate reaches 10%, the image quality improves significantly. As the sampling rate increases further, the differences among the reconstructed images become nearly imperceptible.

To more precisely quantify the reconstruction efficiency of IMCFT, particularly in comparison with static Fourier-SPI with IFT reconstruction, we conducted numerical simulations based on the framework described in Supplementary Note 5. We present IMCFT reconstructions at multiple sampling rates and plot detailed PSNR and SSIM curves. IMCFT performs weighted summation over motion-compensated basis patterns, and when the target is stationary IMCFT reduces to the IFT. Thus, we show IFT reconstructions of a static targe at the same sampling rates together with their PSNR and SSIM curves. The results are shown in Supplementary Fig.15.

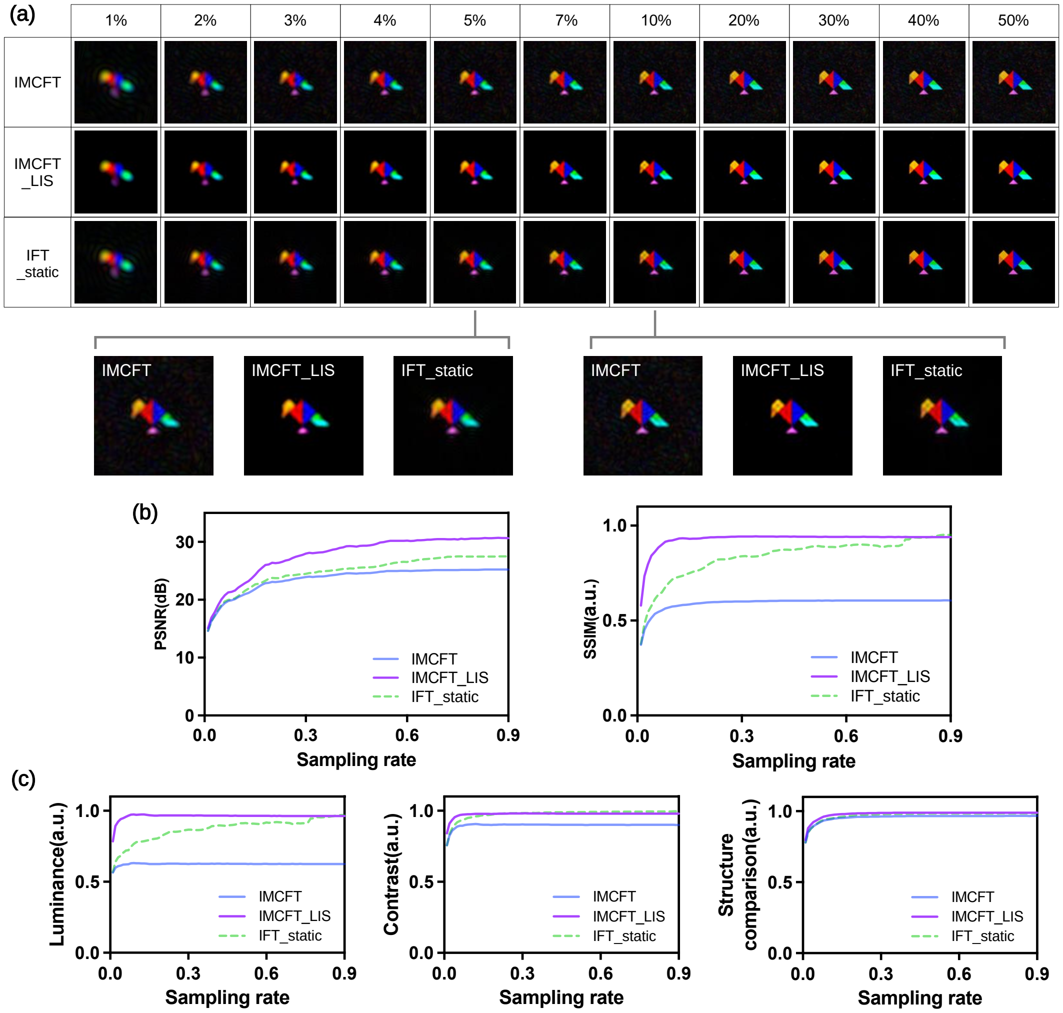

Supplementary Fig. 15: Performance analysis of IMCFT is conducted via numerical simulations across different sampling rates. The IFT reconstruction of a static target (static IFT) serves as the reference, and a Linear intensity stretch (LIS) algorithm is applied for image enhancement. (a) Visual comparison of the reconstructions. (b) PSNR and SSIM results. (c) Results of the three SSIM components, namely luminance, contrast, and structure comparison.

Compared with the previous experimental results, when using true original image as references in the numerical simulations, the PSNR and SSIM of the IMCFT reconstructions are lower. This outcome is expected because the basis patterns are deliberately rotated, which degrades the IMCFT reconstruction performance. Nevertheless, overall trends are similar, and reconstruction using only the first 10% of samples already yields a clear target image.

As shown in the Supplementary Fig. 15 (a), when visually comparing the reconstructions of IMCFT and static IFT, we do not observe noticeable differences. In a visual comparison between IMCFT and static IFT at a sampling rate of 5%, both reconstructions display the characteristic ringing artifacts of Fourier-SPI at low sampling rates. Aside from the relatively more pronounced cluttered background in IMCFT, the reconstruction quality of the target itself is nearly identical. At a sampling rate of 10%, the reconstructed target images from IMCFT and static IFT are nearly indistinguishable when the background is disregarded. Even at a sampling rate of 50%, the two reconstructions differ only marginally and their structural content is almost identical.

By contrast, in Fig. 15 (b) the visual inspection agrees reasonably well with PSNR, and the PSNR curves of IMCFT and static IFT differ only slightly across sampling rates, proving that IMCFT reconstructed a low-noise image[13]. However, the visual impression diverges markedly from SSIM. After a rapid rise,

the SSIM of IMCFT stabilizes at about 0.6 and shows no further increase, leaving a pronounced gap relative to static IFT.

Considering the definition of SSIM, suppose x and y are two nonnegative image signals, the SSIM is composed of three terms: luminance $l(x, y)$, contrast $c(x, y)$, and structure comparison $s(x, y)$, which is defined as follow:

$$\mathrm{SSIM}(x, y) = [l(x, y)]^{\alpha} [c(x, y)]^{\beta} [s(x, y)]^{\gamma}, \tag{27}$$

with

$$l(x, y) = \frac{2\mu_x\mu_y + C_1}{\mu_x^2 + \mu_y^2 + C_1}, \qquad c(x, y) = \frac{2\sigma_x\sigma_y + C_2}{\sigma_x^2 + \sigma_y^2 + C_2}, \qquad s(x, y) = \frac{\sigma_{xy} + C_3}{\sigma_x\sigma_y + C_3}. \tag{28}$$

A common choice sets $\alpha = \beta = \gamma = 1$ and $C_3 = C_2/2$. Under this setting SSIM can simplify to Eq.21.

To investigate the causes of the significant decline in SSIM, we performed numerical simulations for each of its three components individually. The results are shown in Supplementary Fig. 15 (c).

We find that the primary cause of the suboptimal SSIM of IMCFT lies in the luminance term; the luminance of IMCFT reconstructions differs substantially from that of static IFT, whereas the contrast and structure comparison terms perform well, with the former stabilizing around 0.9 and the latter approaching 1. It should be noted that the IMCFT reconstructions exhibit a persistent low-intensity low-frequency blotchy background that disperses target energy; furthermore, we normalized the reconstructions to match the `imshow` display range setting `[]` in MATLAB (`imshow(img, [])`), which inflates the overall brightness of IMCFT images and thereby contributes to the aforementioned SSIM discrepancy. Nevertheless, the strong performance in contrast and structure comparison indicate that IMCFT has potential for rapid target recognition at low sampling rates.

We apply a simple linear intensity stretch which smooth high-intensity regions and suppress low-intensity low-frequency blotchy in the background, thereby mitigating the luminance inflation introduced by normalization and enhancing the image. Visual inspection of IMCFT reconstructions before and after processing indicates that the linear intensity stretch does not alter the structural content of the target image, yet it significantly improves PSNR and SSIM, even outperforming static IFT. Therefore, we believe that IMCFT is effective in capturing the target structure and thereby enabling target recognition. Moreover, linear contrast stretch is computationally lightweight and requires only ~ 0.0002 seconds per application. The linear intensity stretch algorithm is detailed in Algorithm 1. It should be noted that for IMCFT the linear intensity stretch algorithm is not necessary. Although applying this algorithm leads to a noticeable increase in SSIM, the improvement in visual appearance is minimal. In scenarios such as target recognition that focus on structural features, the algorithm offers no practical benefit. Linear intensity stretch becomes a reasonable choice when the aim is to enhance reconstruction quality while retaining a lightweight and simple procedure.

Algorithm 1 Linear intensity stretch

Require: uint8 RGB matrix `image`, stretch factor $\alpha = 1.2$
Ensure: Matrix `image` stretched to [0, 255]

`image` $\leftarrow$ (`image` $- 128) \times \alpha + 128$
Set values below 0 to 0: `image`(`image` $< 0) = 0$
Set values above 255 to 255: `image`(`image` $> 255) = 255$
Convert to unsigned 8-bit integers: `image` = uint8(`image`)
return `image`

SSIM places greater emphasis on comparing structural information between reference and reconstructed images, making it more aligned with perceived visual quality[14, 15]. Based on the SSIM results and the direct visual perception of the reconstructed images, with an approximately 5% sampling, the

IMCFT method can reconstruct images sufficient for reliable target recognition, enabling MC3-SPI to rapidly identify high-speed targets.

Supplementary Note 9: Extend-pixel imaging of a motion target

Our previous experiments successfully captured the motion state of a target moving at the edge of the field of view and reconstructed its complete image, thereby extending the effective optical field of view of the system to some extent. Building on this, we can realize extend-pixel SPI imaging, where the number of pixels in the reconstructed image exceeds that of the SPI patterns, enabling complete reconstruction of a target whose size exceeds the system's field of view. To validate our claim, we conducted numerical simulations and the results are shown in the Supplementary Fig.16.

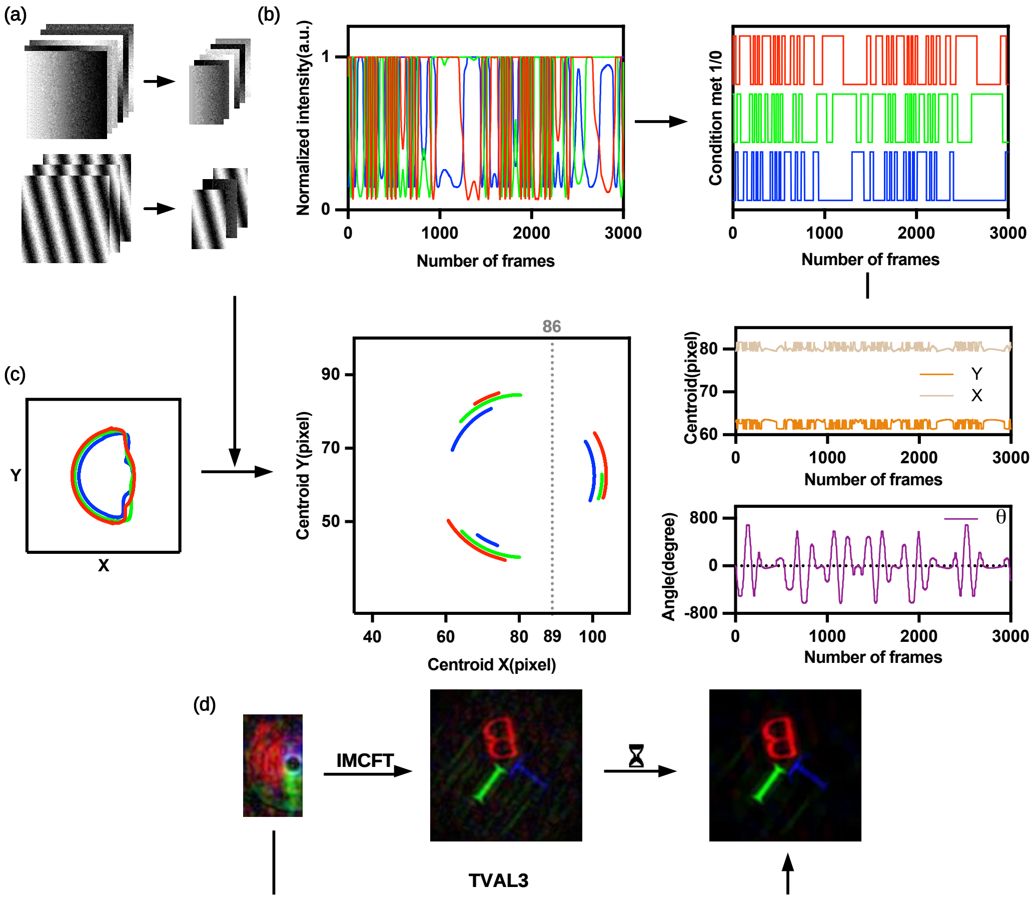

Supplementary Fig. 16: Pattern design for extend-pixel imaging together with motion sensing and motion-compensated imaging results for a target larger than the field of view. In detail, a 128×128 image was reconstructed using 86×50 pixel patterns, with the target measuring approximately 80×80 pixels. (a) Original patterns and real DMD-patterns. (b) Preliminary determination of whether a target channel lies within the field of view based on its intensity. (c) The directly measured trajectory and the trajectory after correction and completion, together with the corresponding target motion information, namely the x and y coordinates and the orientation angle θ. (d) Direct reconstructions, IMCFT extend-pixel reconstructions, and results optimized using TVAL3.

A key to extend-pixel imaging is how to expand the patterns. We adopt a top-down strategy that crops small patterns directly from larger ones, as shown in Supplementary Fig.16(a). Small patterns are used during SPI acquisition, whereas the original-size patterns are used for image reconstruction. Because Fourier patterns permit arbitrary pixel dimensions, an appropriate pixel size can be selected

to accommodate targets of different sizes and reduce additional SPI measurements, making Fourier patterns a suitable choice.

Since the target is larger than the field of view, the complete target cannot be observed and it moves persistently along the field boundary, which matches the motion-sensing approach described in the subsection Extended-FOV tracking and imaging of a cross-boundary motion target in the main text. We conducted numerical simulations of extend-pixel imaging, reconstructing a 128×128 image using 86×50-pixel patterns, and the results are presented in Supplementary Fig.16(b)–(d).

In Supplementary Fig.16(c), we normalize the SPI-measured target channel intensities and apply thresholding to determine whether the corresponding channel lies within the field of view, which guides the validation of the directly computed trajectory. Supplementary Fig.16(c) displays the raw trajectory together with the corrected and completed valid trajectory, and reports the two-dimensional motion information of the target, which we use to perform motion-compensated reconstruction. Supplementary Fig.16(d) presents the reconstruction without motion compensation and without extend-pixel processing, as well as the reconstruction obtained with IMCFT and extend-pixel processing. These results show that we successfully reconstruct the complete image of a target larger than the field of view and that the conventional TVAL3 algorithm effectively refines the reconstruction.

Supplementary Note 10: Target angle estimation in SPI: Multi-channel Multicentroid vs Principal Component Analysis

In SPI, an effective technique for characterizing a rotating object employs geometric moments and principal component analysis (PCA); the major axis of the object can be determined from just six low-order moments[16]. PCA identifies the principal axis of the target by analyzing the variance of its image projections onto axes at multiple orientations. For a target image $I(x, y)$, the raw moments are shown as follows:

$$m_{pq} = \sum_x \sum_y x^p \, y^q \, I(x, y). \tag{29}$$

The centroid of the target (x_c, y_c) can be obtained from the zeroth-order and first-order moments:

$$x_c = \frac{m_{10}}{m_{00}}, \quad y_c = \frac{m_{01}}{m_{00}}. \tag{30}$$

Then calculate the second-order central moments, which measure the spread of the intensity distribution around the centroid:

$$\begin{aligned}
\mu_{20} &= \sum_x \sum_y (x - x_c)^2 \, I(x, y) = m_{20} - x_c m_{10}, \\
\mu_{02} &= \sum_x \sum_y (y - y_c)^2 \, I(x, y) = m_{02} - y_c m_{01}, \\
\mu_{11} &= \sum_x \sum_y (x - x_c)(y - y_c) \, I(x, y) = m_{11} - x_c m_{01} = m_{11} - y_c m_{10}.
\end{aligned} \tag{31}$$

Form the covariance matrix of these moments:

$$\mathbf{M} = \begin{pmatrix} \mu_{20} & \mu_{11} \\ \mu_{11} & \mu_{02} \end{pmatrix}. \tag{32}$$

The principal orientation θ (relative to the x-axis) is the angle of the major eigenvector of $\mathbf{M}$, which simplifies to:

$$\theta = \frac{1}{2}\arctan\Bigl(\frac{2\,\mu_{11}}{\mu_{20}-\mu_{02}}\Bigr). \tag{33}$$

This formula yields θ in radians; multiply by $180/\pi$ to express it in degrees.

To quantitatively compare the performance of our multichannel multi-centroid angle determination method with the PCA-based approach, we conducted data simulations, and the virtual trajectory of the target is presented in Supplementary Material 5. For an identical motion, selecting a different reference point yields a distinct translational trajectory, whereas the relative angular variation remains unchanged. Accordingly, this study evaluates only the angular accuracy. Simulations were performed for three distinct targets, and the corresponding results are presented in Supplementary Fig.17.

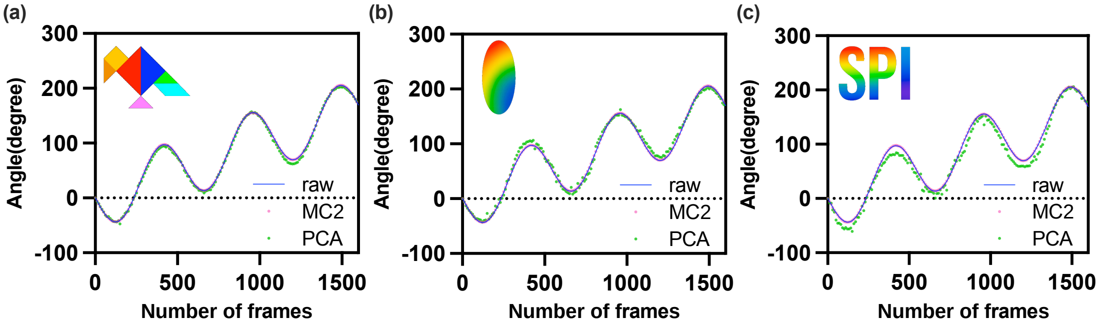

Supplementary Fig. 17: Multichannel multi-centroid (MC2) vs principal component analysis (PCA): angular accuracy. (a) The RMSE of MC2 is 0.2909°. The RMSE of PCA is 3.3903°. (b) The RMSE of MC2 is 0.5015°. The RMSE of PCA is 5.6262°. (c) The RMSE of MC2 is 0.4042°. The RMSE of PCA is 10.5520°.

The multichannel multi-centroid method achieves an angular estimation accuracy that is an order of magnitude higher than that of PCA. The classical definition of image moments is continuous, whereas in the SPI framework both the target images and the patterns are discretized. In the discrete domain, rotation is not rigorously defined, since rotating a discrete image typically yields an approximate and often irreversible transformation. Consequently, PCA founded on geometric moments can only approximate the target's orientation.

In addition, PCA based on geometric moments can, in theory, recover only the principal axis of the target, which means that it cannot determine its absolute orientation, therefore cannot distinguish a 180° flip. This ambiguity can be eliminated in engineering practice by invoking a continuity assumption. As long as the imaging system provides sufficiently high temporal resolution, the detected angles can be assumed to vary smoothly; consequently, when an abrupt change is observed, subsequent angles are corrected by adding or subtracting 180°. Doing so yields a continuous rotational trajectory and allows the full rotational motion of the target to be reconstructed. In Supplementary Fig.17, the approach is applied to correct the angles obtained from PCA, and the algorithmic procedure is presented as Algorithm 2.

Algorithm 2 Angle Continuity Correction for θ_{GM}

Require: A sequence of angles $\theta_{\mathrm{GM}}[1\ldots N]$ in degrees
Ensure: A corrected sequence with no large jumps ($>150°$)

```
for i = 1 to N − 1 do
    if θ_GM[i + 1] − θ_GM[i] > 150 then
        θ_GM[i + 1] ← θ_GM[i + 1] − 180
    end if
    if θ_GM[i + 1] − θ_GM[i] < −150 then
        θ_GM[i + 1] ← θ_GM[i + 1] + 180
    end if
end for
```

As noted above, for orientation estimation within the SPI framework, the multichannel multicentroid method outperforms the PCA approach based on geometric moments. However, SPI is capable of directly retrieving image moments of arbitrary order, eliminating the need for secondary post-processing of images. Geometric moments, especially central moments, can be combined to yield the Hu invariants, which remain unchanged under translation, scaling, and rotation and therefore offer a basis for image analysis[17, 18]. Thus, SPI that exploits geometric moments enables target detection without images and capitalizes on the inherently high throughput and high sensitivity of SPI.

Supplementary Note 11: Discussion and analysis of the sub-pixel motion

A core component of MC3-SPI is motion compensation; however, in practice it cannot be perfect. In addition to the usual errors introduced by noise, systematic errors also arise; for example, our optimized localization method still exhibits approximately one-third pixel localization error, and within each measurement frame there is a small temporal offset between localization and imaging. Therefore, after compensating the dominant target motion, residual small motions often persist and remain imperfectly compensated. This residual motion is typically small in magnitude and sub-pixel at the SPI sampling resolution. We therefore analyze this residual sub-pixel motion within the Fourier-SPI framework, which is best suited to MC3-SPI.

In a continuous model, let the image be $f(\mathbf{r})$ and the small displacement be $\boldsymbol{\delta}$. Define the difference:

$$\Delta(\mathbf{r}) = f(\mathbf{r} - \boldsymbol{\delta}) - f(\mathbf{r}). \tag{34}$$

Denote the Fourier transforms by $F(\boldsymbol{\omega})$ and $\widehat{\Delta}(\boldsymbol{\omega})$. Then,

$$\widehat{\Delta}(\boldsymbol{\omega}) = \left(e^{-j\,\boldsymbol{\omega\delta}} - 1\right) F(\boldsymbol{\omega}) = -\,2j\,e^{-j\,\boldsymbol{\omega\delta}/2}\,\sin\!\left(\frac{\boldsymbol{\omega\delta}}{2}\right) F(\boldsymbol{\omega}), \tag{35}$$

For the small displacement $\boldsymbol{\delta}$,

$$\widehat{\Delta}(\boldsymbol{\omega}) \approx -j\,(\boldsymbol{\omega}\cdot\boldsymbol{\delta})\,F(\boldsymbol{\omega}). \tag{36}$$

Therefore, the effect of a small displacement on the Fourier spectrum exhibits two characteristics. First, its magnitude scales linearly with the small parameter $\boldsymbol{\delta}$, which means that the effect is limited. Second, it acts as a first-order high-pass filter, since the associated factor is proportional to $\boldsymbol{\omega}$.

In summary, in dynamic Fourier-SPI the effect of the sub-pixel displacement of a target is expected to be modest and more pronounced at higher spatial frequencies. Because low-frequency components carry greater weight for perceived image quality, the theoretical impact of sub-pixel displacement on final image quality is limited. To further quantify this effect, we performed numerical simulations in which the target underwent continuous random sub-pixel motion throughout SPI sampling, where the sub-pixel shifts were uniformly distributed in $[-0.5, 0.5]$ pixels; the results are shown in Supplementary Fig. 18.

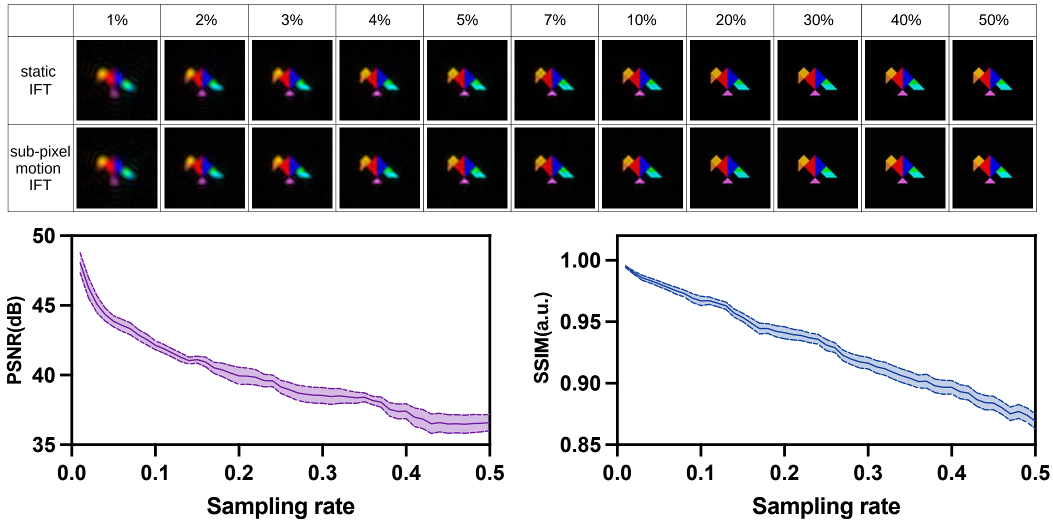

Supplementary Fig. 18: Analysis of the impact of sub-pixel motion on the final imaging quality of Fourier-SPI across different sampling rates. The upper panel presents a visual comparison of the reconstructions; the lower panel reports PSNR and SSIM with respect to a static IFT reference at the corresponding sampling rate, using 20 repeats with standard deviations shown as error bands.

Numerical simulations show that sub-pixel motion has a nearly imperceptible effect on Fourier-SPI reconstructions, which agrees with our previous analysis. And from the standpoint of motion-induced ghosting, sub-pixel shifts contribute only weakly to the final weighted-sum reconstruction, particularly for the main body of the target. Quantitatively, PSNR and SSIM decrease when the sampling ratio increases, yet the absolute values remain high overall, which is consistent with our earlier analysis that the influence of sub-pixel motion is more concentrated in the high-frequency components and is limited overall. We therefore conclude that in MC3-SPI the effect of sub-pixel errors is limited and can generally be neglected.

Finally, it should be noted that Hadamard-SPI shares the same distribution of information weights across spatial frequencies as Fourier-SPI, so the theoretical impact of sub-pixel errors should likewise be small.

Supplementary Note 12: Discussion and analysis of the limitations of MC3-SPI

Although MC3-SPI can efficiently sense target motion and orientation, its applicability is constrained by several fundamental assumptions. On the one hand, some limitations arise from our method itself, which inherently requires the intensity distributions across channels to break central symmetry so that the channel centroids are measurably separated for rotation sensing; on the other hand, intrinsic constraints of the SPI system preclude accurate target capture and imaging in interfering environments such as multi-object scenes or strong backgrounds.

However, owing to the flexibility of MC3-SPI, customized designs can be incorporated within its framework to remain effective in the aforementioned specific scenarios.

Limitations for centrosymmetric targets

Our method relies on multichannel observations: the target exhibits different intensity distributions across channels, resulting in measurable offsets between the channel centroids and the global centroid. By analyzing the directional angles between each channel centroid and the global centroid, the instantaneous orientation of the target can be inferred. However, if the target's signal distribution is centro-symmetric

across all channels, the channel centroids coincide with the global centroid, providing no separable centroid information and rendering orientation estimation infeasible. Consequently, MC3-SPI functions reliably only when the signal distribution in at least one channel breaks central symmetry.

However, perfectly centrosymmetric objects are rare, and finer channel configurations can be adopted, for example extending from RGB to multispectral or even hyperspectral channels, to reveal object asymmetries. Moreover, even when an object is perfectly centrosymmetric in a single optical dimension, multiple optical dimensions can be jointly exploited, such as wavelength and polarization, to determine the wavelength centroid and the polarization centroid and thereby infer the target's rotational information. Therefore, our method is theoretically effective across a wide range of objects.

When the object is completely symmetrical, it is worth mentioning that the localization of the target position remains accurate. This suggests that, after applying translational compensation, the angular variation of the target during SPI acquisition may be estimated from the quality of the reconstructed images [19]. Furthermore, the proposed IMCFT provides a fast and effective reconstruction approach to support this estimation. Despite, the introduction of optimization algorithms incurs a prohibitive computational cost, causing real-time performance to be lost entirely.

In summary, our target angle sensing method does have limitations, yet we maintain that it nonetheless retains appreciable value.

Limitations when multiple objects are present in the field of view

Conventional SPI exhibits a typical global sensing mechanism: Each measurement integrates information from the entire field of view, and without prior knowledge of target location, it cannot focus on specific objects. Existing SPI-based localization methods typically extract only a "global centroid", which is an aggregate quantity rather than the spatial coordinates of an individual target. When multiple targets or strong background interference are present in the field, the global centroid is influenced by all signal sources, rendering localization of the target of interest ineffective. Let pixels (x_i, y_i) for $i = 1, \ldots, n$ have weights (masses) m_i. The global centroid $(\bar{x}, \bar{y})$ is defined as follows:

$$\bar{x} = \sum_{i=1}^{n} w_i \, x_i \quad \text{and} \quad \bar{y} = \sum_{i=1}^{n} w_i \, y_i, \quad w_i = \frac{m_i}{\sum_{j=1}^{n} m_j}. \tag{37}$$

When there is only a single target in the field of view, the global centroid defined by the above equation is evidently equivalent to the centroid of that target. However, when multiple targets are present in the field of view, the global centroid becomes equivalent to the intensity-weighted average of the individual target centroids.

Assume that n objects are present within the field of view. Let the centroids of objects $(\bar{x}_i, \bar{y}_i)$ for $i = 1, \ldots, n$ have weights (masses) M_i. The global centroid output by the SPI location method $(\bar{x}, \bar{y})$ is defined as follows:

$$\bar{x} = \sum_{i=1}^{n} w_i \, \bar{x}_i \quad \text{and} \quad \bar{y} = \sum_{i=1}^{n} w_i \, \bar{y}_i, \quad w_i = \frac{M_i}{\sum_{j=1}^{n} M_j}. \tag{38}$$

It follows that any non-target object present in the field of view "pulls" the SPI localization result, displacing it from the desired position. When such interfering objects are stationary, their influence can be entirely eliminated by a simple procedure. With prior knowledge of the precise positions and sizes of these static objects (a reasonable assumption since the objects are static), we set the corresponding regions of the SPI measurement patterns to zero, thereby rendering the interferences invisible to the SPI system. A straightforward numerical simulation was carried out to validate this idea, as shown in the Supplementary Fig.19.

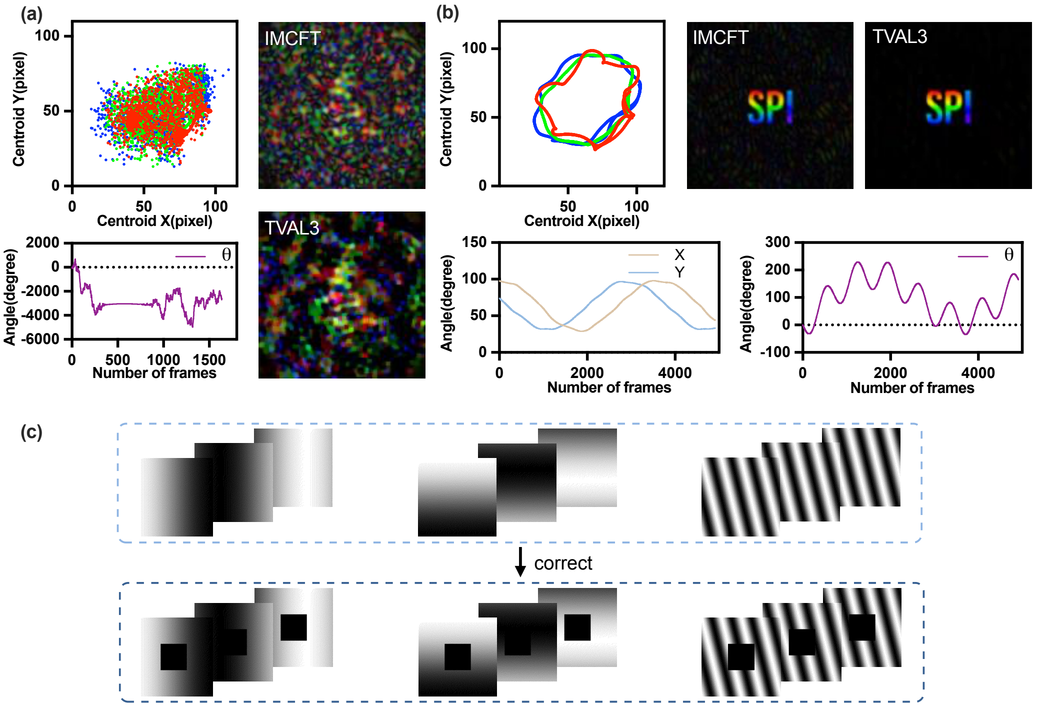

Supplementary Fig. 19: Numerical simulation results of MC3-SPI in the presence of a strongly interfering object that flickers randomly at the center of the field of view, together with the pattern optimization strategy used to suppress the interferer and the corresponding MC3-SPI results. (a) Direct motion sensing and imaging results in the presence of a flickering strong interferer. (b) Motion sensing and imaging results obtained with optimized patterns in the presence of a strongly interfering flickering object. (c) Pattern optimization method that sets the regions corresponding to interfering object in the patterns to zero, thereby eliminating its influence entirely.

The initial intensity of the interfering object is set to twice that of the target, and in each simulated measurement, each of its color channels is individually multiplied by a random factor to emulate random flickering. The interfering object is placed at the center of the field of view. This constitutes severe interference and leads to complete failure of the method, as shown in Supplementary Fig.19 (a).

To eliminate the influence of non-target objects, we set the corresponding regions of the patterns to zero, as shown in the Supplementary Fig.19 (c). In this way, SPI completely ignores any variations in those regions without requiring additional modifications. As a result, our method accurately captures the target's motion information and reconstructs a high-quality image of the target, as illustrated in the Supplementary Fig.19 (b).

However, when the interfering objects in the scene also move unpredictably, the above strategy becomes ineffective. More sophisticated approaches are required to isolate the target signal, for example, exploiting its distinctive emission wavelength or polarization characteristics.

Limitations in the presence of a nonzero background

Another scenario involves a target moving against a substantial background. Although this situation resembles the multi-object case, the crucial difference is that the target obscures a small region of the background. Consequently, the global centroid determined by the SPI system becomes an intensity-weighted average of the unoccluded background and the target centroid. When addressing this problem, the occluded portion of the background can be modeled as an object with negative weight, thereby reducing the background–occlusion scenario to the multi-object case.

Let the background centroid be denoted $(\bar{x}_b, \bar{y}_b)$ with weight M_b, the target centroid $(\bar{x}_t, \bar{y}_t)$ with weight m_t, and the centroid of the background region occluded by the target $(\bar{x}'_t, \bar{y}'_t)$ with weight m'_t. The

global centroid output by the SPI location method $(\bar{x}, \bar{y})$ is defined as follows:

$$\begin{aligned} \bar{x} &= \frac{m_t}{m_t + M_b - m_t'}\bar{x}_t + \frac{M_b}{m_t + M_b - m_t'}\bar{x}_b + \frac{-m_t'}{m_t + M_b - m_t'}\bar{x}_t' \\ \text{and} \quad \bar{y} &= \frac{m_t}{m_t + M_b - m_t'}\bar{y}_t + \frac{M_b}{m_t + M_b - m_t'}\bar{y}_b + \frac{-m_t'}{m_t + M_b - m_t'}\bar{y}_t'. \end{aligned} \tag{39}$$

We now concentrate on the case of a static, invariant background. Adopting the reasonable prior that the background has been fully characterized, the initial step is to subtract its contribution from every SPI measurement. It is important to note that the occluded part of the background is never captured by SPI measurements; therefore, subtracting the complete background effectively introduces a negative artifact that coincides with the target. This negatively weighted artifact is effectively equivalent to a positively weighted one located at a negative position, and its presence exerts a pronounced influence on the system. Numerical simulations were conducted for this scenario and the results are presented in Supplementary Fig.20.

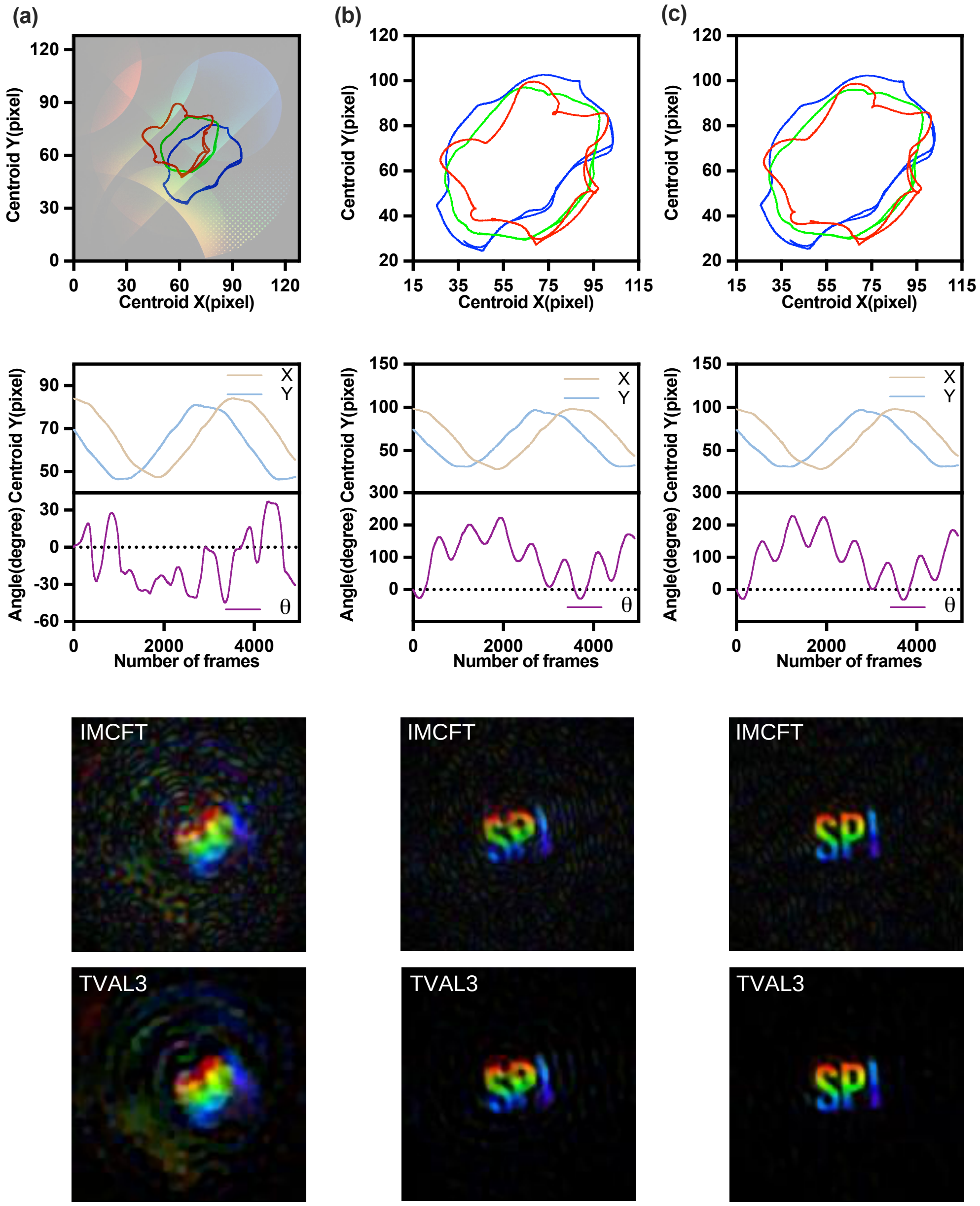

Supplementary Fig. 20: Numerical simulation in the presence of a non-negligible static background, with motion sensing and imaging results at three representative stages of the MC3-SPI optimization process. (a) Motion sensing and imaging results in the presence of background. (b) Motion sensing and imaging results with direct subtraction of the complete background. (c) Motion sensing and imaging results with iterative application of Algorithm 3 to appropriately eliminate background effects.

In the simulation, we set the total background intensity equal to that of the target. Although the background intensity remained relatively low, it still caused our method to fail, as shown in the Supplementary Fig.20 (a).

Supplementary Fig.20 (b) shows the result obtained by subtracting the full background contribution from the SPI measurements. The background intensity is dispersed through the field of view; the region masked by the target should have only a minor weight. Its negative weight amplifies its effect, producing severe distortions in the reconstructed target image.

Accordingly, we use the IMCFT result in Fig.20 (b) as a reference, generate a mask by thresholding, and apply it to the background to avoid the negatively weighted artifact introduced by subtracting the full background from the SPI measurements. The newly corrected SPI measurements are then used for the

IMCFT reconstruction. By iterating these steps an appropriate number of times, an undistorted target image can be obtained, as shown in Fig.20 (c). The algorithmic procedure is presented as Algorithm 3.

Algorithm 3 Iterative background correction

Require:
Location patterns P_l, imaging patterns P_{im},
Raw measurements $\{R_l, R_{im}\}$,
Fullcolour background matrix *background*.

Ensure: Get the corrected measurements $\{R'_l, R'_{im}\}$

Initialize $mask$ to all zeros
Initialize $\Delta x, \Delta y, \Delta\theta$ to all zeros
for iteration $\ell = 1$ **to** 3 **do**
  $mask \leftarrow 1 - mask$
  for frame number $i = 1$ **to** num **do**
    $mask_i \leftarrow \text{circshift}(\text{imrotating}(mask,\ -\Delta\theta(i)),\ [\Delta y(i), \Delta x(i)])$
    $R'_l(\cdot) \leftarrow R_l(\cdot) - P_l(\cdot)\langle mask_i \odot background\rangle$
    $R'_{im}(\cdot) \leftarrow R_{im}(\cdot) - P_{im}(\cdot)\langle mask_i \odot background\rangle$
  end for
  $\Delta x, \Delta y, \Delta\theta \leftarrow \text{MC2}(R'_l)$
  $U \leftarrow \text{IMCFT}(R'_{im},\ [\Delta x, \Delta y, \Delta\theta])$
  $mask \leftarrow \text{im2double}(\text{im2gray}(U))$
  $mask(mask < 0.2) \leftarrow 0$
  $mask \leftarrow mask \circledast ones(13 - 2\ell)$
  $\text{mask}(\text{mask} \neq 0) \leftarrow 1$
end for

Notably, the proposed IMCF method enables rapid reconstruction of two-dimensional targets undergoing compound motion and is particularly well suited for loop-structured implementations.

Additionally, in the Algorithm 3 we convolve the image with all-ones kernels of different sizes to blur and thereby expand the object, ensuring that the regions set to one in the mask completely cover the target. In this case, we prefer leaving residual background to over-subtracting it; the former introduces peripheral noise around the target, whereas the latter yields a negatively weighted artifact with a strong impact.

However, our method is effective only when the background remains static. It will inevitably fail if the background undergoes complex variations or if multiple targets enter the scene simultaneously. We require an efficient approach to completely eliminate background contributions. Axial discrimination using the photon time of flight is a promising option. Once the target distance is determined, signals originating from other ranges can be rejected in full. Because the background is often not coplanar with the target, this strategy removes background interference regardless of how the background varies.

References

[1] Zibang Zhang, Jiaquan Ye, Qiwen Deng, and Jingang Zhong. Image-free real-time detection and tracking of fast moving object using a single-pixel detector. *Optics express*, 27(24):35394–35401, 2019.

[2] Shijian Li, Xu-Ri Yao, Wei Zhang, Yeliang Wang, and Qing Zhao. Tracking and fast imaging of a moving object via fourier modulation. *Physical Review Applied*, 22(4):044007, 2024.

[3] Zibang Zhang, Xueying Wang, Guoan Zheng, and Jingang Zhong. Fast fourier single-pixel imaging via binary illumination. *Scientific reports*, 7(1):12029, 2017.

[4] Robert W Floyd. An adaptive algorithm for spatial gray-scale. In *Proc. Soc. Inf. Disp.*, volume 17, pages 75–77, 1976.

[5] Zhen-Yu Liang, Zheng-Dong Cheng, Yan-Yan Liu, Kuai-Kuai Yu, and Yang-Di Hu. Fast fourier single-pixel imaging based on sierra–lite dithering algorithm. *Chinese physics B*, 28(6):064202, 2019.

[6] Heyan Meng, Yuan Gao, Xuhong Wang, Xianye Li, Lili Wang, Xian Zhao, and Baoqing Sun. Quantum dot-enabled infrared hyperspectral imaging with single-pixel detection. *Light: Science & Applications*, 13(1):121, 2024.

[7] Linbin Zha, Dongfeng Shi, Jian Huang, Kee Yuan, Wengweng Meng, Wei Yang, Runbo Jiang, Yafeng Chen, and Yingjian Wang. Single-pixel tracking of fast-moving object using geometric moment detection. *Optics express*, 29(19):30327–30336, 2021.

[8] Zibang Zhang, Xueying Wang, Guoan Zheng, and Jingang Zhong. Hadamard single-pixel imaging versus fourier single-pixel imaging. *Optics Express*, 25(16):19619–19639, 2017.

[9] Gene H Golub and Charles F Van Loan. *Matrix computations*. JHU press, 2013.

[10] David L Donoho and Michael Elad. Optimally sparse representation in general (nonorthogonal) dictionaries via ℓ_1 minimization. *Proceedings of the National Academy of Sciences*, 100(5):2197–2202, 2003.

[11] Michael Elad. *Sparse and redundant representations: from theory to applications in signal and image processing*. Springer Science & Business Media, 2010.

[12] Thomas Maitre, Elie Bretin, Romain Phan, Nicolas Ducros, and Michaël Sdika. Dynamic single-pixel imaging on an extended field of view without warping the patterns. In *International Conference on Medical Image Computing and Computer-Assisted Intervention*, pages 275–284. Springer, 2024.

[13] I˙ smail Avcıbas¸, Bu¨ lent Sankur, and Khalid Sayood. Statistical evaluation of image quality measures. *Journal of Electronic imaging*, 11(2):206–223, 2002.

[14] Zhou Wang, Alan C Bovik, Hamid R Sheikh, and Eero P Simoncelli. Image quality assessment: from error visibility to structural similarity. *IEEE transactions on image processing*, 13(4):600–612, 2004.

[15] Zhou Wang and Alan C Bovik. Mean squared error: Love it or leave it? a new look at signal fidelity measures. *IEEE signal processing magazine*, 26(1):98–117, 2009.

[16] Leping Xiao, Jianyu Wang, Xintong Liu, Xinxing Lei, Zuoqiang Shi, Lingyun Qiu, and Xing Fu. Single-pixel imaging of a randomly moving object. *Optics Express*, 30(22):40389–40400, 2022.

[17] Ming-Kuei Hu. Visual pattern recognition by moment invariants. *IRE transactions on information theory*, 8(2):179–187, 1962.

[18] Yudong Zhang, Shuihua Wang, Ping Sun, and Preetha Phillips. Pathological brain detection based on wavelet entropy and hu moment invariants. *Bio-medical materials and engineering*, 26(1_suppl):S1283–S1290, 2015.

[19] Shuming Jiao, Mingjie Sun, Yang Gao, Ting Lei, Zhenwei Xie, and Xiaocong Yuan. Motion estimation and quality enhancement for a single image in dynamic single-pixel imaging. *Optics express*, 27(9):12841–12854, 2019.
