# Supplementary material for: Multichannel multicentroid motion-compensated single pixel imaging of a 2D arbitrarily moving rigid-body target
Source: Commun Eng. 2026 Feb 25;5:61. doi: 10.1038/s44172-026-00619-2 (PMC13046804; doi:10.1038/s44172-026-00619-2)
Supplement: Supplementary file 3 — Description of Additional Supplementary Files [file 44172_2026_619_MOESM3_ESM.pdf]

Description of Additional Supplementary Files

The work includes two supplementary videos, each comprising multiple segments that illustrate the target's motion in various experiments described in the main manuscript. The schematic representations of the supplementary videos are shown below:

- **Supplementary Movie 1**

This is a video consists of four parts, corresponding to the four tracking and imaging experiments in the main manuscript.

In the video, the first part shows the real-time IMCT reconstruction of the target, demonstrating the process of the target transitioning from blurred to clear. The last three parts use TVAL3 reconstruction results, focusing on the target's different motion states.

Since the DMD's deflection frequency varies across the four experiments, the number of frames captured per second for the target differs, at 16.67Hz, 500Hz, 27.78Hz, and 222.2Hz, respectively. We standardized the video frame rate to 222fps and combined the videos for easier viewing.

- **Supplementary Movie 2**

This is a video consists of three parts, corresponding to the three displays of results in the Extended-FOV tracking and imaging experiments in the main manuscript.

In the video, the first part shows the full actual motion of the target, with the gray box indicating the FOV boundary; the second part mainly demonstrates the real-time IMCFT reconstruction process of the target within the FOV; and the third part employs the TVAL3 reconstruction result to primarily display the target's real-time motion state.

You will notice that the target's trajectory in the video appears discontinuous, exhibiting jumps due to our motion-state filtering in the extended-FOV imaging. The video is rendered at 393 fps to limit its duration to approximately 30 s.
